# KHDRBS1 regulates the pentose phosphate pathway and malignancy of GBM through SNORD51-mediated polyadenylation of ZBED6 pre-mRNA

**DOI:** 10.1038/s41419-024-07163-x

**Published:** 2024-11-08

**Authors:** Xiaoyu Liu, Xiaobai Liu, Weiwei Dong, Ping Wang, Libo Liu, Lu Liu, Tiange E, Di Wang, Yang Lin, Hongda Lin, Xuelei Ruan, Yixue Xue

**Affiliations:** 1 Department of Neurobiology, School of Life Sciences, China Medical University, Shenyang, 110122 China; 2 Key Laboratory of Neuro-oncology in Liaoning Province, Shenyang, 110004 China; 3 Department of Neurosurgery, Shengjing Hospital of China Medical University, Shenyang, 110004 China

**Keywords:** CNS cancer, RNA metabolism

## Abstract

Glioblastoma is one of the most common and aggressive primary brain tumors. The aberration of metabolism is the important character of GBM cells and is tightly related to the malignancy of GBM. We mainly verified the regulatory effects of KHDRBS1, SNORD51 and ZBED6 on pentose phosphate pathway and malignant biological behavior in glioblastoma cells, such as proliferation, migration and invasion. KHDRBS1 and SNORD51 were upregulated in GBM tissues and cells. But ZBED6 had opposite tendency in GBM tissues and cells. KHDRBS1 may improve the stability of SNORD51 by binding to SNORD51, thus elevating the expression of SNORD51. More importantly, SNORD51 can competitively bind to WDR33 with 3’UTR of ZBED6 pre-mRNA which can inhibit the 3’ end processing of ZBED6 pre-mRNA, thereby inhibiting the expression of ZBED6 mRNA. ZBED6 inhibited the transcription of G6PD by binding to the promoter region of G6PD. Therefore, the KHDRBS1/SNORD51/ZBED6 pathway performs an important part in regulating the pentose phosphate pathway to influence malignant biological behavior of GBM cells, providing new insights and potential targets for the treatment of GBM.

## Introduction

As a primary brain tumor, glioblastoma multiforme (GBM) is the most typical type. Although the multi-modality combination of therapy for glioblastoma has achieved remarkable achievements, the prognosis of patients with glioblastoma is still very poor [1, 2]. High amounts of reactive oxygen species (ROS) are found in cancer cells. For maintaining the redox homeostasis of cancer cells, pentose phosphate pathway (PPP) which can produce reducing agents such as nicotinamide adenine dinucleotide phosphate (NADPH) performs an important part in this process [3, 4]. In GBM cells, upregulation of nuclear factor erythroid 2-related factor 2 (Nrf2) promotes the expression of glucose-6-phosphate dehydrogenase (G6PD) and transketolase (TKT) to inhibit ROS-induced apoptosis [4]. The PPP, as the important pathway of cell energy metabolism, has become an important target of tumor pathogenesis and treatment.

RNA-binding proteins (RBPs) participates in alternative splicing, polyadenylation, intracellular localization and translation of mRNA for influencing tumorigenesis and growth [5]. For example, macrophages with overexpressed RBP-J inhibit glioma growth by secreting circRNA BTG2 [6]. YTH domain family, member 2 (YTHDF2) inhibits the MYC-driven cell death in breast cancer cells [7]. KH RNA binding domain containing, signal transduction associated 1 (KHDRBS1) is engaged in signal transduction, transcription, RNA metabolism and apoptosis [8]. Researches have indicated that KHDRBS1 is upregulated in colon cancer, prostate cancer, kidney cancer and colorectal cancer [9]. KHDRBS1 is upregulated in GBM cells and promotes the growth of glioblastoma [10].

Small nucleolar RNAs (snoRNAs) are the class of non-coding RNAs that regulate post-transcriptional modification, methylation, and pseudouridylation of ribosomal RNAs (rRNAs) by forming small nucleolar riboprotein complexes with nucleolar proteins [11]. Several studies have demonstrated that snoRNAs regulate biological cancer processes [12]. For example, SNORA42 knockdown inhibits proliferation and self-renewal of non-small cell lung cancer cells, and SNORD126 promotes the growth of hepatocellular and colon cancer cells by activating the phosphatidylinositol 3-kinase (PI3K)/protein kinase B (PKB/AKT) signaling pathway [13]. SNORD51 has been confirmed to participate in the 2’-O-methylation modification of the protein coding region of peroxidasin (Pxdn) mRNA, thereby regulating the level of ROS in cells [14]. However, the mechanism and functions of snoRNAs have not been verified to regulate the proliferation, migration and invasion of GBM cells by affecting intracellular ROS.

As the zinc finger protein, zinc finger bed-type containing 6 (ZBED6) regulates the transcription of downstream target genes. ZBED6 affects the growth and development of mammalian skeletal muscle and internal organs by inhibiting the expression of IGF2 [15–17]. ZBED6 regulates the balance between cell proliferation and differentiation in pancreatic β-cells and affects the production of cellular ROS by regulating mitochondrial activity [18, 19]. In colorectal cancer, silencing of ZBED6 promotes the proliferation of RKO cells [16].

G6PD, the rate-limiting enzymes of the PPP, catalyzes the generation of NADPH from NADP [20]. G6PD promotes the proliferation of renal cell carcinoma and ectopically transplanted human melanoma cells in mouse models by regulating the signal transducer and activator of transcription 3 (STAT3) signaling pathway. Besides, the overactivation of G6PD promotes the proliferation of esophageal squamous cell carcinoma [21–23]. G6PD is significantly elevated in GBM tissues and G6PD promotes the development of GBM [24]. G6PD performs an important part in tumor growth, PPP and oxidative stress and is a key factor in regulating metabolic reprogramming of cancer cells [25].

## Results

### KHDRBS1 was upregulated in GBM tissues and cells, promoted the PPP and the malignant biological behavior of GBM cells

**Fig. 1:**
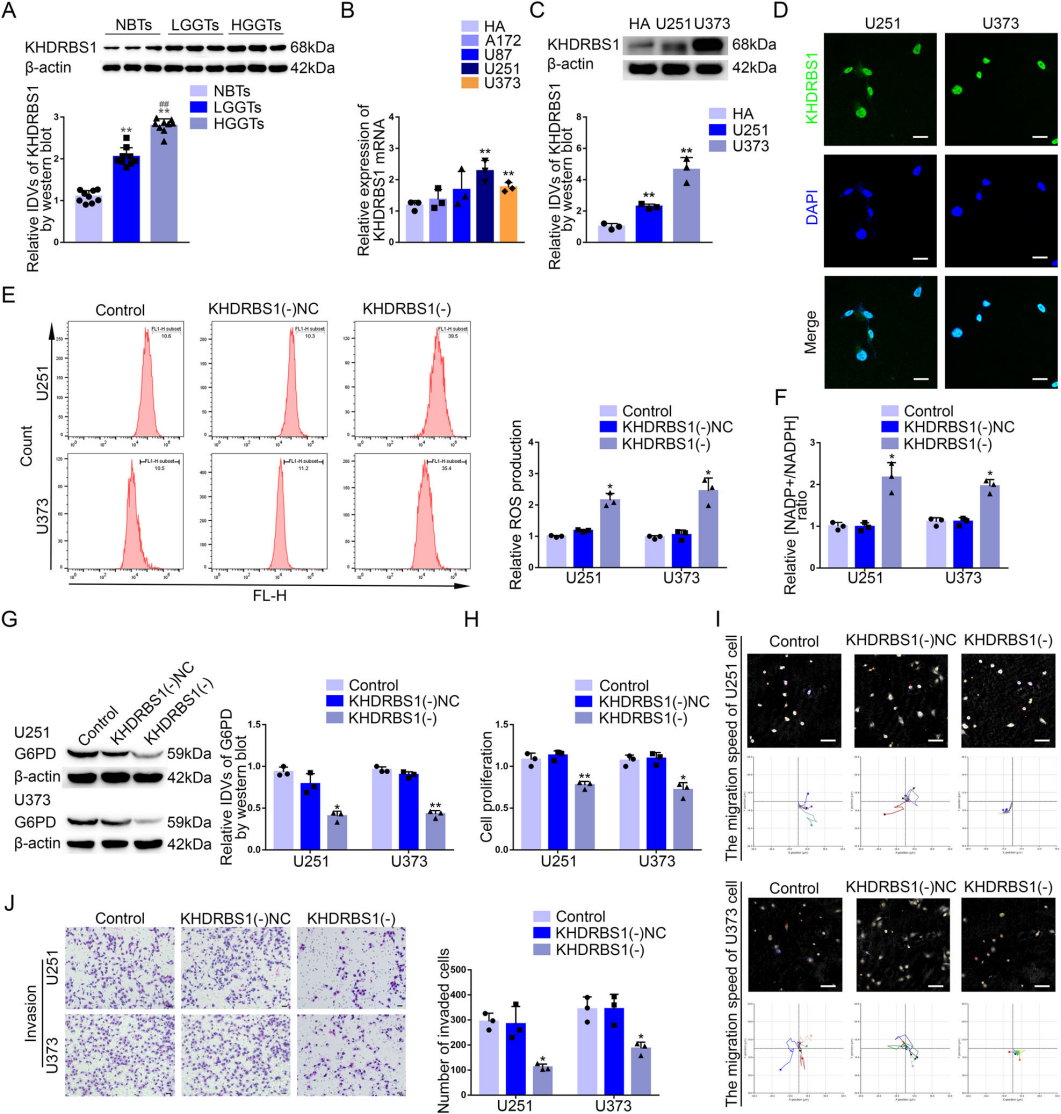
KHDRBS1 was upregulated in GBM tissues and cells, promoting the PPP and biological behavior of GBM cells. **A** The protein expression level of KHDRBS1 was detected in NBTs, LGGTs and HGGTs. Data are presented as the mean ± SD (*n* = 9, each group). ^**^P < 0.01 versus NBTs group; ^##^*P* < 0.01 versus LGGTs group. **B** The mRNA expression level of KHDRBS1 in four GBM lines (A172, U87, U251, U373). Data are presented as the mean ± SD (*n* = 3, each group). ^**^P < 0.01 versus HA group. **C** The protein expression level of KHDRBS1 was detected in human astrocytes (HA) and GBM lines (U251 and U373) by western blot. Data are presented as the mean ± SD (*n* = 3, each group). ^**^P < 0.01 versus HA group. **D** The location of KHDRBS1 (green) in the cytoplasm and nuclear fractions (blue) of U251 and U373 cells was detected by immunofluorescence (IF). Nuclei were labeled with DAPI. Scale bars: 20 µm. **E**, **F** The effects of KHDRBS1 knockdown on pentose phosphate pathway in U251 and U373 cells were verified by detecting intracellular ROS level and the relative ratio of NADP + /NADPH. Data are presented as the mean ± SD (*n* = 3, each group). ^*^P < 0.05 versus KHDRBS1(−)NC group. **G** G6PD protein level after KHDRBS1 knockdown in U251 and U373 cells was detected by western blot. Data are presented as the mean ± SD (*n* = 3, each group). ^*^P < 0.05 versus KHDRBS1(−)NC group. **H** The effect of KHDRBS1 knockdown on the proliferation of U251 and U373 cells was detected by CCK-8 assay. Data are presented as the mean ± SD (*n* = 3, each group). ^*^P < 0.05 versus KHDRBS1(−)NC group; ^**^P < 0.01 versus KHDRBS1(−)NC group. **I** The effect of KHDRBS1 knockdown on cell migration of U251 and U373 cells was analyzed by Hstudio M4 system. Scale bars: 100 µm. **J** The effect of KHDRBS1 knockdown on the capacity for invasion in U251 and U373 cells was detected by Transwell method. Scale bars: 50 µm. Data are presented as the mean ± SD (*n* = 3, each group). ^*^P < 0.05 versus KHDRBS1(−)NC group.

Based on GSE90598 dataset obtained from the Gene Expression Omnibus (GEO), we analyzed the RNA sequencing results of GBM tissues and normal human brain tissues for differential gene analysis. The top 200 differentially expressed genes were selected according to log2-fold change (Supplementary Fig. 1A). Then, the top 50 upregulated protein-coding genes were screened to made the Sangkey plot (Supplementary Fig. 1B). We ranked the genes related to mRNA metabolic process according to their areas under the curve (AUC) and made the receiver operating characteristic (ROC) curves of them based on GSE90598 dataset. KHDRBS1 (the area under the curve, AUC = 0.972) had stronger sensitivity and specificity of GBM than other genes (Supplementary Fig. 1C). Furthermore, the Cancer Genome Atlas Program (TCGA) database showed that KHDRBS1 was significantly associated with poor prognosis of glioma (Supplementary Fig. 1D). The analysis of TCGA database indicated both mutation of IDH and 1p/19q codeletion were correlated with KHDRBS1 expression (Supplementary Table 1). Compared with normal brain tissues, the results showed that both mRNA and protein expression levels of KHDRBS1 was significantly increased and positively correlated with pathological grades (Supplementary Fig. 1E, Fig. 1A). Compared to the human astrocytes (HA) group, the mRNA expression of KHDRBS1 increased significantly in U251 and U373 groups. U251 and U373 cell lines were selected for further research (Fig. 1B). We also discovered that the protein level of KHDRBS1 was significantly upregulated in GBM cells compared with HA (Fig. 1C). Immunofluorescence (IF) assay showed that KHDRBS1 was distributed in the nucleus of GBM cells (Fig. 1D).

Based on the results of relative ROS production and relative NADP + /NADPH ratio assays, KHDRBS1 knockdown significantly inhibited the PPP (Fig. 1E, F). We also found that KHDRBS1 knockdown significantly decreased the protein level of G6PD and it had no effect on the protein level of PGD (Fig. 1G, Supplementary Fig. 10C). Furthermore, KHDRBS1 knockdown significantly inhibited the proliferation of GBM cells based on the results of Cell Counting Kit-8 (CCK-8) assay (Fig. 1H). Through M4-Studio system, we observed that the migration of GBM cells was decreased after KHDRBS1 knockdown (Fig. 1I). Transwell assay revealed that the invasion of GBM cells was significantly decreased after KHDRBS1 knockdown (Fig. 1J).

### SNORD51 was upregulated in GBM tissues and cells, promoted the PPP and malignant biological behavior of GBM cells

**Fig. 2:**
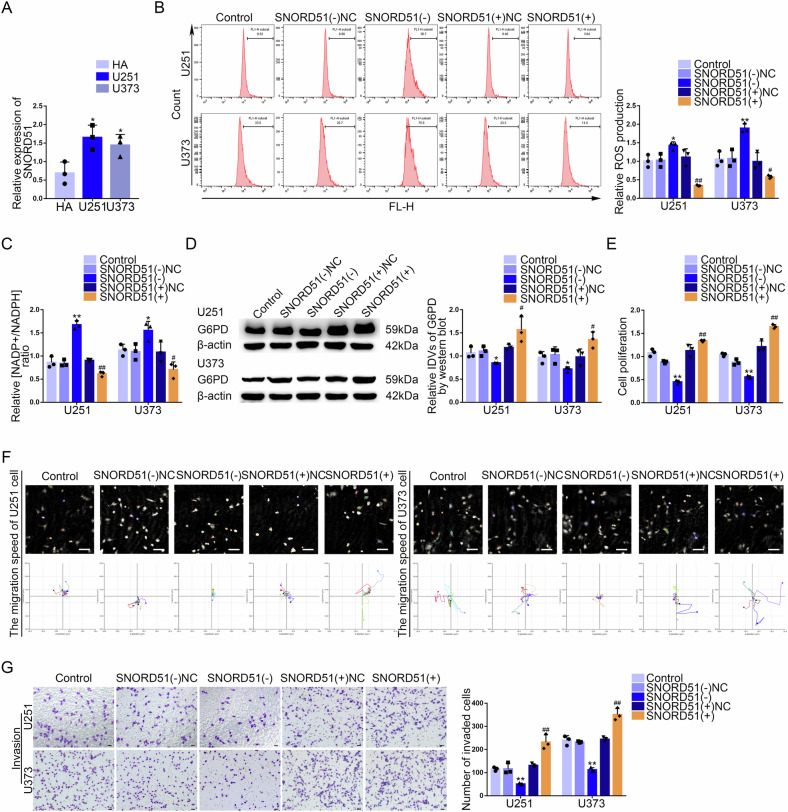
SNORD51 was upregulated in GBM tissues and cells, promoting the PPP and biological behavior of GBM cells. **A** The relative expression of SNORD51 was analyzed in HA, U251 and U373 cells by qRT-PCR. Data are presented as the mean ± SD (*n* = 3, each group). ^*^P < 0.05 vs HA group; ^**^P < 0.01 vs HA group. **B**, **C** The effect of SNORD51 on pentose phosphate pathway was analyzed by intracellular ROS level and relative ratio of NADP + /NADPH in U251 and U373 cells with altered expression of SNORD51. Data are presented as the mean ± SD (*n* = 3, each group). ^**^P < 0.01 vs SNORD51(^−^)NC group; ^##^*P* < 0.01 vs SNORD51( + )NC group. **D** The protein level of G6PD was detected by western blot in U251 and U373 cells with altered expression of SNORD51. ^*^P < 0.05 vs SNORD51(−)NC group; ^#^*P* < 0.05 vs SNORD51( + )NC group. **E** The effect of SNORD51 on cell proliferation of U251 and U373 cells was analyzed by CCK-8 assay. Data are presented as the mean ± SD (*n* = 3, each group). ^**^P < 0.01 vs SNORD51(−)NC group; ^##^*P* < 0.01 vs SNORD51( + )NC group. **F** The effect of SNORD51 on cell migration of U251 and U373 cells was analyzed by Hstudio M4 system. Scale bars: 100 µm. **G** The effect of SNORD51 on cell invasion of U251 and U373 cells was analyzed by Transwell method. Scale bars: 50 µm. Data are presented as the mean ± SD (*n* = 3, each group). ^**^P < 0.01 vs SNORD51(−)NC group; ^##^*P* < 0.01 vs SNORD51( + )NC group.

Based on GSE100675 dataset, we made the co-expression correlation heatmap of KHDRBS1 and 18 snoRNAs selected by ranking their correlation coefficients in GBM. The Volcano plot was made to screen out 5 upregulated snoRNAs in GBM (Supplementary Fig. 2B). By drawing the Venn diagram, we got 2 snoRNAs that both were upregulated and closely related to KHDRBS1 in GBM (Supplementary Fig. 2C). The selected snoRNAs were analyzed by RNA Interactome Database (RNAInter), and it was found that SNORD51 might interact with KHDRBS1 (Supplementary Fig. 2D). Compared to KHDRBS1(−)NC group, we observed that the expression of SNORD51 was significantly decreased in KHDRBS1(−) group and the expression of SNORD78 was not changed (Supplementary Fig. 2E). Thus, SNORD51 was chosen as the research target. SNORD51 was significantly upregulated in GBM tissues and positively correlated with pathological grades (Supplementary Fig. 2F). Compared to the HA group, the mRNA expression of SNORD51 was significantly increased in U251 and U373 groups (Fig. 2A). SNORD51 knockdown inhibited the PPP but SNORD51 overexpression had the opposite effect (Fig. 2B, C). The protein level of G6PD was significantly decreased after SNORD51 knockdown (Fig. 2D). In addition, we observed that SNORD51 knockdown inhibited the proliferation, invasion and migration of GBM cells. However, SNORD51 overexpression promoted the proliferation, invasion and migration of GBM cells (Fig. 2E–G).

### KHDRBS1 regulated the PPP and malignant biological behavior of GBM cells through SNORD51

**Fig. 3:**
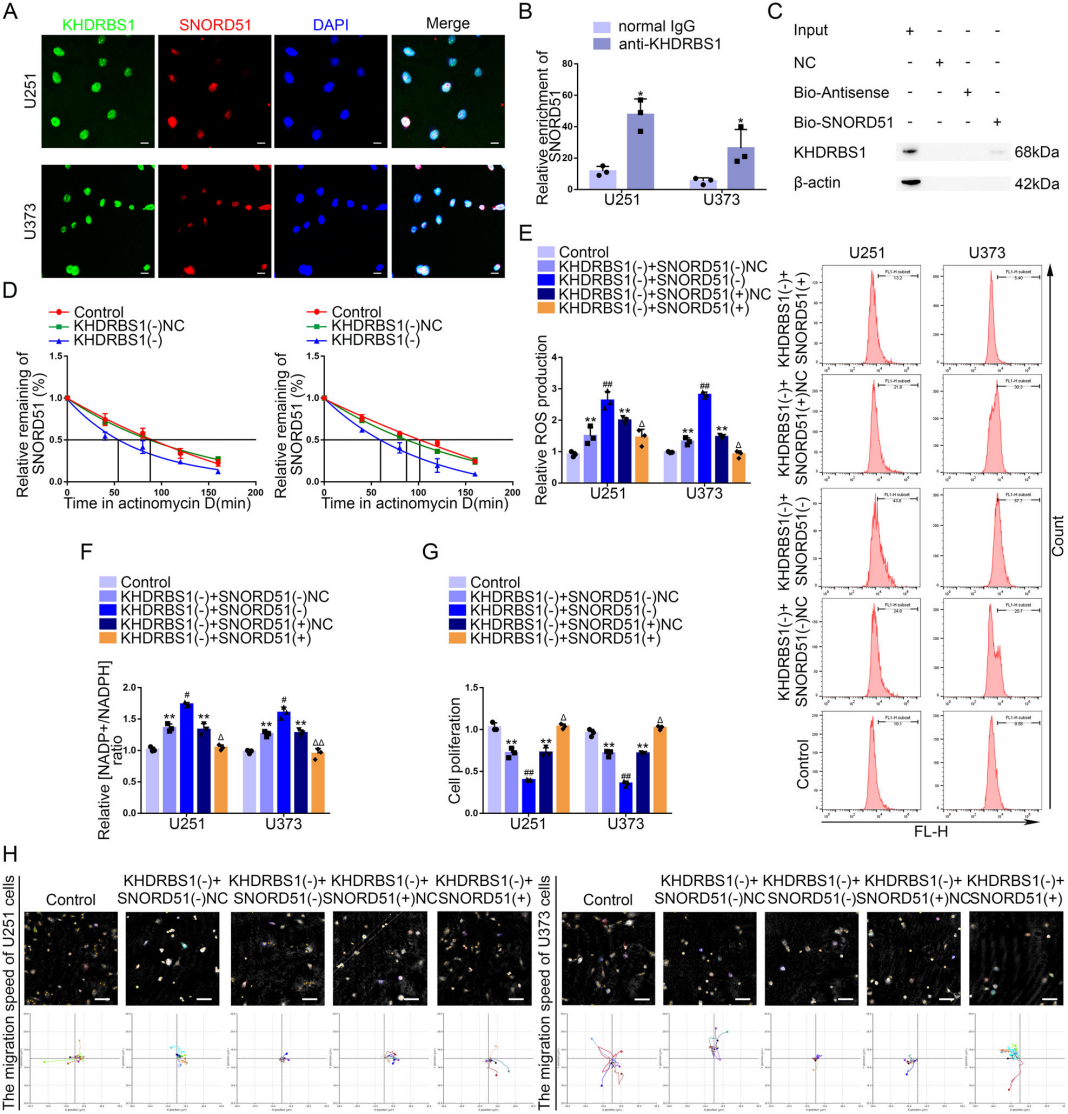
KHDRBS1 enhanced the stability of SNORD51 by binding to SNORD51 promoting the PPP and biological behavior of GBM cells. **A** The location of SNORD51 (red) and KHDRBS1 (green) in the cytoplasm and nuclear fractions (blue) of U251 and U373 cells was detected by IF and FISH. Nuclei were labeled with DAPI. Scale bars: 20 µm. **B** An enrichment of SNORD51 in KHDRBS1 immunoprecipitated samples via RNA immunoprecipitation (RIP) assay. Relative enrichment was measured by quantitative real-time PCR. Data are presented as the mean ± SD (*n* = 3, each group). ^*^P < 0.05 versus normal IgG group. **C** RNA pull-down assay followed by western blot confirmed specific associations of KHDRBS1 with biotinylated-SNORD51. **D** The effect of KHDRBS1 knockdown on the half-life of SNORD51 was measured by RT-qPCR after actinomycin **D** treated in U251 and U373 cells. Data are presented as the mean ± SD (*n* = 3, each group). **E**, **F** The effect of SNORD51 on pentose phosphate pathway was analyzed by intracellular ROS level and relative ratio of NADP + /NADPH in U251 and U373 cells. Data are presented as the mean ± SD (*n* = 3, each group). ^**^P < 0.01 versus Control group; ^#^*P* < 0.05 versus KHDRBS1(−) + SNORD51(−)NC group; ^##^*P* < 0.01 versus KHDRBS1(−) + SNORD51(−)NC group; ^Δ^*P* < 0.05 versus KHDRBS1(−) + SNORD51( + )NC group; ^ΔΔ^*P* < 0.01 versus KHDRBS1(−) + SNORD51( + )NC group. **G** The effect of KHDRBS1 and SNORD51 on cell proliferation of U251 and U373 cells was analyzed by CCK-8 assay. Data are presented as the mean ± SD (*n* = 3, each group). ^**^P < 0.01 versus Control group; ^##^*P* < 0.01 versus KHDRBS1(−) + SNORD51(−)NC group; ^Δ^*P* < 0.05 versus KHDRBS1(−) ^+^ SNORD51( + )NC group. **H** The combined effects of KHDRBS1 and SNORD51 on cell migration of U251 and U373 cells was analyzed by Hstudio M4 system. Scale bars: 100 µm.

To elucidate the relationship between KHDRBS1 and SNORD51, IF and fluorescence in situ hybridization (FISH) assays showed that KHDRBS1 and SNORD51 were distributed in the nucleus (Fig. 3A). Next, RNA Pull-down and RNA immunoprecipitation (RIP) assays confirmed the interaction between KHDRBS1 and SNORD51 (Fig. 3B, C). To further elucidate the mechanism by which KHDRBS1 promoted the expression of SNORD51 in GBM cells, the half-life of SNORD51 was analyzed by quantitative real-time PCR (qRT-PCR). As shown in Fig. 3D, the half-life of SNORD51 in GBM cells was significantly shortened after KHDRBS1 knockdown. These results suggested that KHDRBS1 may bind SNORD51 to increase the stability of SNORD51 and elevate its expression.

SNORD51 rescued the inhibitory effects on the PPP exerted by KHDRBS1 knockdown (Fig. 3E, F). SNORD51 overexpression reversed the inhibition of protein level of G6PD induced by KHDRBS1 knockdown (Supplementary Fig. 3A). Besides, SNORD51 overexpression reversed the inhibition of proliferation, migration and invasion of GBM cells induced by KHDRBS1 knockdown (Fig. 3G, H, Supplementary Fig. 3B).

### ZBED6 was downregulated in GBM tissues and cells, inhibited the PPP and the malignant biological behavior of GBM cells

**Fig. 4:**
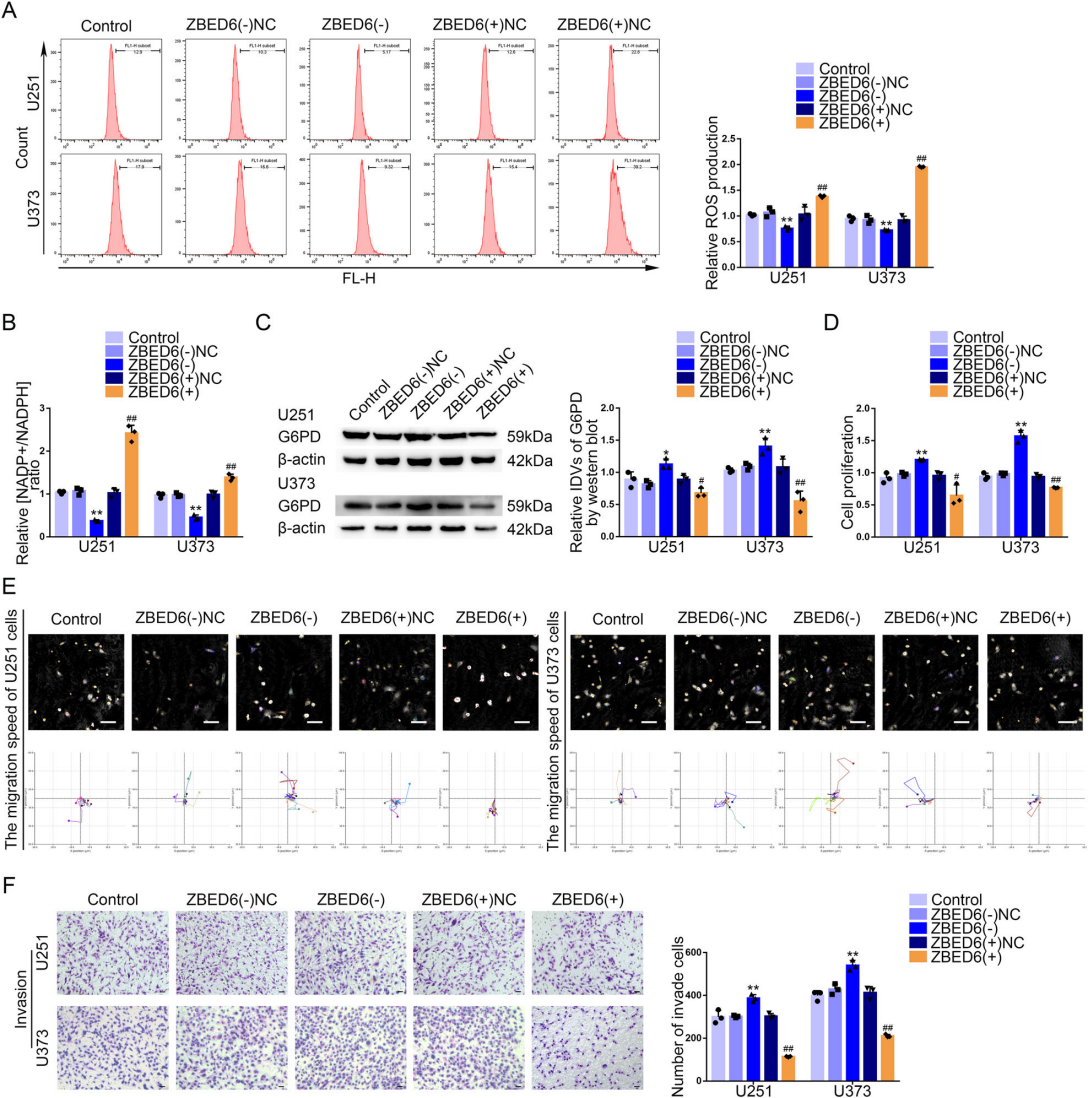
ZBED6, as a tumor suppressor, inhibited the PPP and biological behavior of GBM cells. **A**, **B** The effect of ZBED6 on pentose phosphate pathway was analyzed by intracellular ROS level and relative ratio of NADP + /NADPH in U251 and U373 cells with altered expression of ZBED6. Data are presented as the mean ± SD (*n* = 3, each group). ^**^P < 0.01 versus ZBED6(−)NC group; ^##^*P* < 0.01 versus ZBED6( + )NC group. **C** The protein level of G6PD was detected by western blot in U251 and U373 cells with altered expression of ZBED6. ^*^P < 0.05 vs ZBED6(−)NC group;^**^P < 0.01 vs ZBED6(−)NC group; ^#^*P* ^<^ 0.05 vs ZBED6( + )NC group; ^##^*P* < 0.01 vs ZBED6( + )NC group. **D** The effect of ZBED6 on cell proliferation of U251 and U373 cells was analyzed by CCK-8 assay. Data are presented as the mean ± SD (*n* = 3, each group). ^*^P < 0.05 versus ZBED6(−)NC group; ^**^P < 0.01 versus ZBED6(−)NC group; ^##^*P* < 0.01 versus ZBED6( + )NC group. **E** The effect of ZBED6 on cell migration of U251 and U373 cells was analyzed by Hstudio M4 system. Scale bars: 100 µm. **F** The effect of ZBED6 on cell invasion of U251 and U373 cells was analyzed by Transwell method. Scale bars: 50 µm. Data are presented as the mean ± SD (*n* = 3, each group). ^**^P < 0.01 vs SNORD51(−)NC group; ^##^*P* < 0.01 vs SNORD51( + )NC group.

The SNORD51(−) and SNORD51(−)NC groups GBM cells were analyzed for differential expressed genes by mRNA sequencing, and top 40 differentially expressed protein coding genes were selected to draw heatmaps according to LogFC (Supplementary Fig. 4A). By ranking LogFC, top 10 upregulated protein coding genes were selected to make a Venn diagram in GBM cells after the knockdown of SNORD51 and results showed that ZBED6 and TMEM132C were upregulated in GBM cells with the knockdown of SNORD51 (Supplementary Fig. 4B). Compared to SNORD51(−)NC group, we observed that the mRNA expression level of ZBED6 was significantly increased in SNORD51(−) group and the mRNA expression level of TMEM132C was not significantly changed (Supplementary Fig. 4C). Survival analysis of GBM patients showed that low expression of ZBED6 suggested a poor prognosis (Supplementary Fig. 4D). ROC curve analysis showed that the AUC of ZBED6 was 0.704 (Supplementary Fig. 4E). Compared with normal brain tissues, the results showed that protein expression level of ZBED6 was significantly decreased and negatively correlated with pathological grades. Compared to the human astrocytes (HA) group, the protein expression level of ZBED6 decreased significantly in U251 and U373 groups (Supplementary Fig. 4F, G). ZBED6 knockdown significantly inhibited the PPP but ZBED6 overexpression had opposite effects (Fig. 4A, B). The protein level of G6PD significantly decreased after ZBED6 overexpression (Fig. 4C). In addition, we observed that ZBED6 overexpression inhibited the proliferation, invasion and migration of GBM cells. However, ZBED6 knockdown promoted the proliferation, invasion and migration of GBM cells (Fig. 4D-F).

### SNORD51 affected the 3’end processing of ZBED6 pre-mRNA by competitively binding to WDR33

**Fig. 5:**
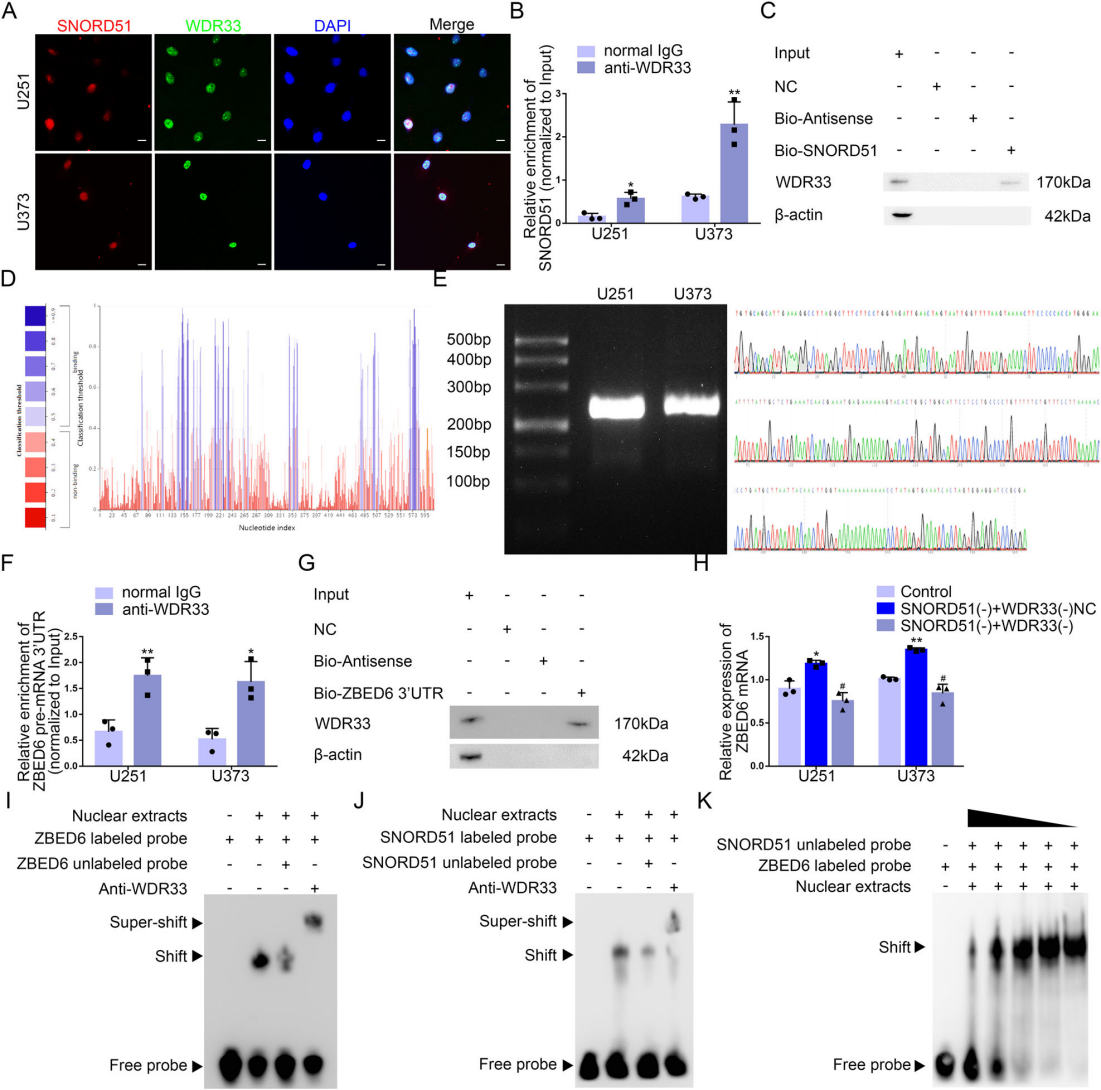
SNORD51 regulated the 3’end polyadenylation of ZBED6 pre-mRNA through competitive binding of WDR33 with 3’UTR of ZBED6 pre-mRNA. **A** The distribution of SNORD51 (red), WDR33 (green) and nuclear fractions (blue) of U251 and U373 cells was detected by IF and FISH. Nuclei were labeled with DAPI. Scale bars: 20 µm. **B** An enrichment of SNORD51 in WDR33 immunoprecipitated samples via RIP assay. Relative enrichment was measured by quantitative real-time PCR. Data are presented as the mean ± SD (*n* = 3, each group).^*^P < 0.05 versus normal IgG group; ^**^P < 0.01 versus normal IgG group. **C** RNA pull-down assay followed by western blot confirmed specific associations of WDR33 with biotinylated-SNORD51. **D** According to the RNAInter Database, the result showed that the binding sites of WDR33 existed in the 3’ UTR of ZBED6 pre-mRNA. **E** 3’ RACE PCR amplified full length 3’ UTR of ZBED6 followed by agarose gel electrophoresis confirmed that there was only one polyadenylation product. The sequence of product was detected by Sanger sequencing. **F** An enrichment of 3’UTR of ZBED6 pre-mRNA in WDR33 immunoprecipitated samples via RIP assay. Relative enrichment was measured by quantitative real-time PCR. Data are presented as the mean ± SD (*n* = 3, each group).^*^P < 0.05 versus normal IgG group; ^**^P < 0.01 versus normal IgG group. **G** RNA pull-down assay followed by western blot confirmed specific associations of WDR33 with biotinylated-3’UTR of ZBED6 pre-mRNA. **H** The reverse effect of WDR33 on SNORD51 in term of mRNA expression of ZBED6 in U251 and U373 cells. Data are presented as the mean ± SD (*n* = 3, each group). ^*^P < 0.05 versus Control group; ^**^P < 0.01 versus Control group; ^#^*P* < 0.05 versus SNORD51(+) + WDR33( + )NC group. **I** Electrophoretic Mobility Shift Assay (EMSA) experiment proved that biotin-labeled 3’UTR of ZBED6 pre-mRNA probe could bind to WDR33. **J** EMSA experiment proved that biotin-labeled SNORD51 probe could bind to WDR33. **K** The biotin-labeled 3’UTR of ZBED6 pre-mRNA probe could The 3’UTR probe labeled with biotin ZBED6pre-mRNA could competitively bind WDR33 with the non-biotin labeled SNORD51 probe.

Based on SNOOPY database, SNORD51 did not have complementary sequence of ZBED6 pre-mRNA (Supplementary Fig. 5A). The result suggested that SNORD51 did not complementarily pair with the sequence of ZBED6 pre-mRNA to regulate the expression of ZBED6. Recent studies had shown that the core subunits of CPSF complexes such as CPSF100, WDR33 and Fip1 recognized the polyadenylation signal by AAUAAA. snoRNAs regulated the 3’end processing of pre-mRNAs by competitively binding with subunits of CPSF complexes. The result of sequence analysis of SNORD51 found that SNORD51 contained AAUAAA hexamer sequence which suggested SNORD51 could bind to the mentioned above subunits. According to RNAInter and RPISeq database, WDR33 could bind to SNORD51 (Supplementary Fig. 5B, C). IF and FISH assays showed that SNORD51 and WDR33 were distributed in the nucleus (Fig. 5A). Next, RNA Pull-down and RIP assays confirmed the interaction between SNORD51 and WDR33 (Fig. 5B, C). Based on RNAInter and RPISeq, WDR33 could bind to 3’UTR of ZBED6 pre-mRNA (Supplementary Fig. 6A, Fig. 5D). The polyadenylation of the 3’UTR of ZBED6 pre-mRNA was detected by 3’ Rapid Amplification of cDNA Ends (3’RACE) followed by agarose gel electrophoresis and Sanger sequencing, the results showed that there was a polyadenylation site in the 3’UTR of ZBED6 pre-mRNA (Supplementary Fig. 7A–C, Fig. 5E). RNA Pull-down and RIP assays confirmed the interaction between WDR33 and 3’UTR of ZBED6 pre-mRNA (Fig. 5F, G). We overexpressed WDR33 on the basis of the construction of GBM cells with SNORD51 overexpression, and detected the expression level of ZBED6 mRNA by qRT-PCR. It was found the expression level of ZBED6 mRNA significantly decreased in GBM cells after SNORD51 overexpression, and WDR33 overexpression reversed the inhibition of the expression level of ZBED6 mRNA induced by SNORD51 overexpression (Fig. 5H). These results suggest that both SNORD51 and 3’ UTR of ZBED6 mRNA may bind to WDR33, thereby participating in the regulation of polyadenylation of ZBED6 pre-mRNA. In order to clarify the regulatory relationship among SNORD51, 3’ UTR of ZBED6 mRNA and WDR33, we designed specific primers containing T7 promoter to synthesize non-biotin-labeled SNORD51 probe and biotin-labeled 3’ UTR of ZBED6 pre-mRNA probe through in vitro transcription followed by sanger sequencing (Supplementary Fig. 8A). The results of competitive gel mobility shift assay showed that SNORD51 competitively binds to WDR33 with 3’ UTR of ZBED6 mRNA (Fig. 5I–K). These results suggested that SNORD51 competitively binds to WDR33 with 3’ UTR of ZBED6 mRNA and inhibits the polyadenylation of 3’ UTR of ZBED6 mRNA by WDR33, thus downregulating the expression of ZBED6 mRNA in GBM cells.

### ZBED6 regulated the expression of G6PD by binding its promoter region

**Fig. 6:**
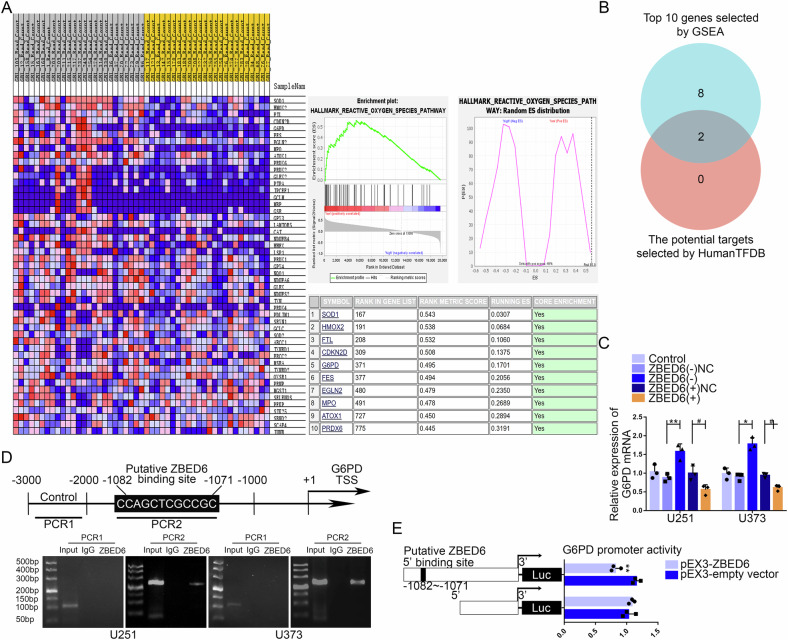
ZBED6 regulated the expression of G6PD by binding its promoter region. **A** By ranking the enrichment scores, top 10 reactive oxygen species pathway genes related to the expression of ZBED6 were screened based on gene set enrichment analysis (GSEA). **B** Based on human transcription factors database (HumanTFDB), the Venn diagram was made to screen the downstream regulatory targets of ZBED6. **C** qRT-PCR assay verified the effect of ZBED6 expression on the expression mRNA level of G6PD in GBM cells. Data are presented as the mean ± SD (*n* = 3, each group). ^*^P < 0.05 versus ZBED6(−)NC group; ^**^P < 0.01 versus ZBED6(−)NC group; ^#^*P* < 0.05 versus ZBED6( + )NC group. **D** Chromatin immunoprecipitation (ChIP) assay confirmed the binding interaction between ZBED6 and the promoter region of G6PD genes. **E** Schematic diagram of luciferase reporter construction and G6PD relative luciferase activity measured in cells cotransfected with the G6PD promoter (or G6PD promoter-deleted putative ZBED6 binding site) and pEX3-empty vector or pEX3-ZBED6. ^**^P < 0.01 versus pEX3-empty vector.

To clarify how ZBED6 affects the ROS in GBM cells and regulates the proliferation, migration and invasion of GBM cells, the gene set enrichment analysis of ZBED6 was performed on glioma samples obtained from GSE263588 dataset. The results showed that ZBED6 was closely related to the gene set of ROS pathway. According to enrichment scores, top 10 genes of ROS pathway were selected, which were related to ZBED6 in glioma samples (Fig. 6A). According to HumanTFDB, the Venn diagram was made to select the potential downstream targets of ZBED6 from genes mentioned above. The result showed that G6PD and PRDX6 could be regulated by ZBED6 (Fig. 6B). G6PD was the rate-limiting enzyme in the PPP and maintained redox balance in tumor cells by generating NADPH. Therefore, G6PD was selected as the research target. The qRT-PCR was used to verify the change on the mRNA expression level of G6PD in GBM cells. Compared to ZBED6(−)NC group, the results showed that the mRNA expression level of G6PD was significantly increased in ZBED6(−) group. And the mRNA expression level of G6PD was significantly decreased in ZBED6(+) group compared to the ZBED6( + )NC group (Fig. 6C). ChIP experiments were applied to verify that ZBED6 could bind directly to the G6PD promoter region. G6PD PCR primers were designed upstream and downstream of the predicted ZBED6 binding site, while a negative control of PCR primers was designed at least 1000 bp upstream of the predicted ZBED6 binding site (Fig. 6D). Dual luciferase reporter gene assays were performed by constructing G6PD promoter region wild-type and mutant vectors to clarify the ZBED6 binding site. The results showed that the luciferase activity was significantly downregulated in HEK293T cells co-transfected with pEX3-ZBED6 and G6PD promoter region wild-type vectors compared to that co-transfected with pEX3-ZBED6 and G6PD promoter region mutant vectors (Fig. 6E).

### SNORD51 regulated the PPP and malignant biological behavior of GBM cells through ZBED6

**Fig. 7:**
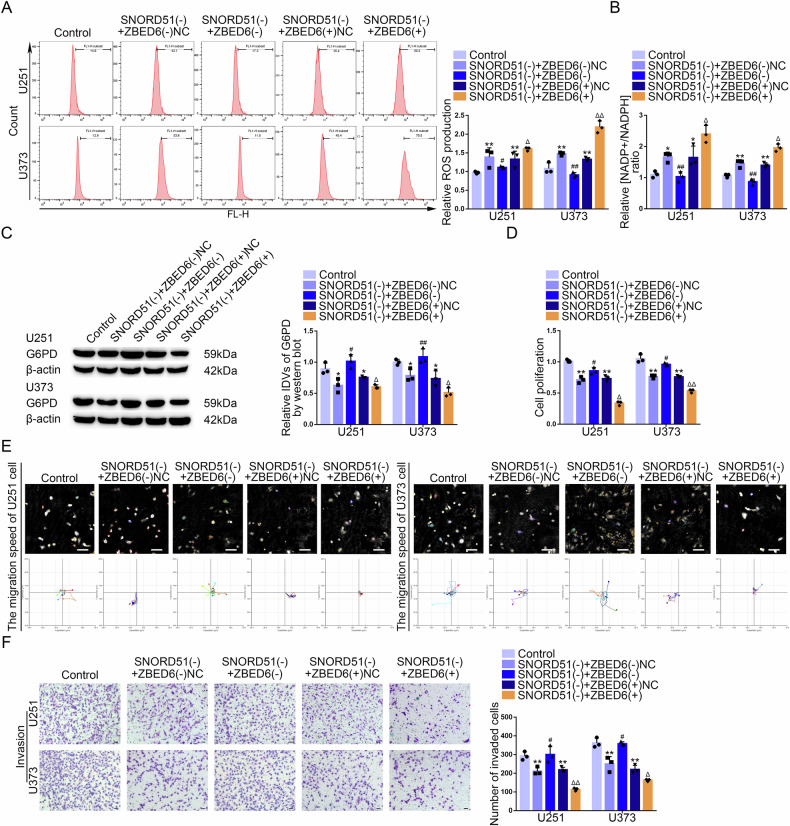
SNORD51 mediated the PPP and the biological behavior of GBM cells through affecting ZBED6. **A**, **B** The reverse effects of SNORD51 on ZBED6 in terms of pentose phosphate pathway were analyzed by intracellular ROS level and relative ratio of NADP + /NADPH in U251 and U373 cells. Data are presented as the mean ± SD (*n* = 3, each group). ^*^P < 0.05 versus Control group; ^**^P < 0.01 versus Control group; ^#^*P* < 0.05 versus SNORD51(−) + ZBED6(−)NC group; ^##^*P* < 0.01 versus SNORD51(−) + ZBED6(−)NC group; ^Δ^*P* < 0.05 versus SNORD51(−) + ZBEDD6( + )NC group; ^ΔΔ^*P* < 0.01 versus SNORD51(−) + ZBED6( + )NC group. **C** The reverse effect of SNORD51 on ZBED6 in term of protein level of G6PD was analyzed by western blot in U251 and U373 cells. Data are presented as the mean ± SD (*n* = 3, each group). ^*^P < 0.05 versus Control group; ^#^*P* < 0.05 versus SNORD51(−) + ZBED6^(^-)NC group; ^##^*P* < 0.01 versus SNORD51(−) ^+^ ZBED6(−)NC group; ^Δ^*P* < 0.05 versus SNORD51(−) + ZBEDD6( + )NC group. **D** The reverse effect of SNORD51 on ZBED6 in term of cell proliferation in U251 and U373 cells was analyzed by CCK-8 assay. Data are presented as the mean ± SD (*n* = 3, each group). ^**^P < 0.01 versus Control group; ^#^*P* < 0.05 versus SNORD51(−) + ZBED6(−)NC group; ^Δ^*P* < 0.05 versus SNORD51(−) + ZBED6( + )NC group. **E** The reverse effect of SNORD51 on ZBED6 in term of cell migration in U251 and U373 cells was analyzed by Hstudio M4 system. Scale bars: 100 µm. **F** The reverse effect of SNORD51 on ZBED6 in term of cell invasion in U251 and U373 cells was analyzed by Transwell method. Scale bars: 50 µm. Data are presented as the mean ± SD (*n* = 3, each group). ^**^P < 0.01 versus Control group; ^#^*P* < 0.05 versus SNORD51(−) + ZBED6(−)NC group; ^Δ^*P* < 0.05 versus SNORD51(−) + ZBEDD6( + )NC group; ^ΔΔ^*P* < 0.01 versus SNORD51(−) + ZBEDD6( + )NC group.

In current study, we found that ZBED6 knockdown reversed the inhibition of the PPP induced by SNORD51 knockdown in GBM cells (Fig. 7A, B). The results of western blot revealed that ZBED6 knockdown reversed the inhibition of the protein expression of G6PD induced by SNORD51 knockdown (Fig. 7C). ZBED6 knockdown reversed the inhibition of the proliferation, migration and invasion induced by SNORD51 knockdown in GBM cells (Fig. 7D–F). In conclusion, SNORD51 regulate the PPP and malignant biological behavior of GBM cells through ZBED6.

### Simultaneous knockdown of KHDRBS1 and SNORD51 combined with the overexpression of ZBED6 significantly inhibited tumor growth and prolonged the survival of nude mice

**Fig. 8:**
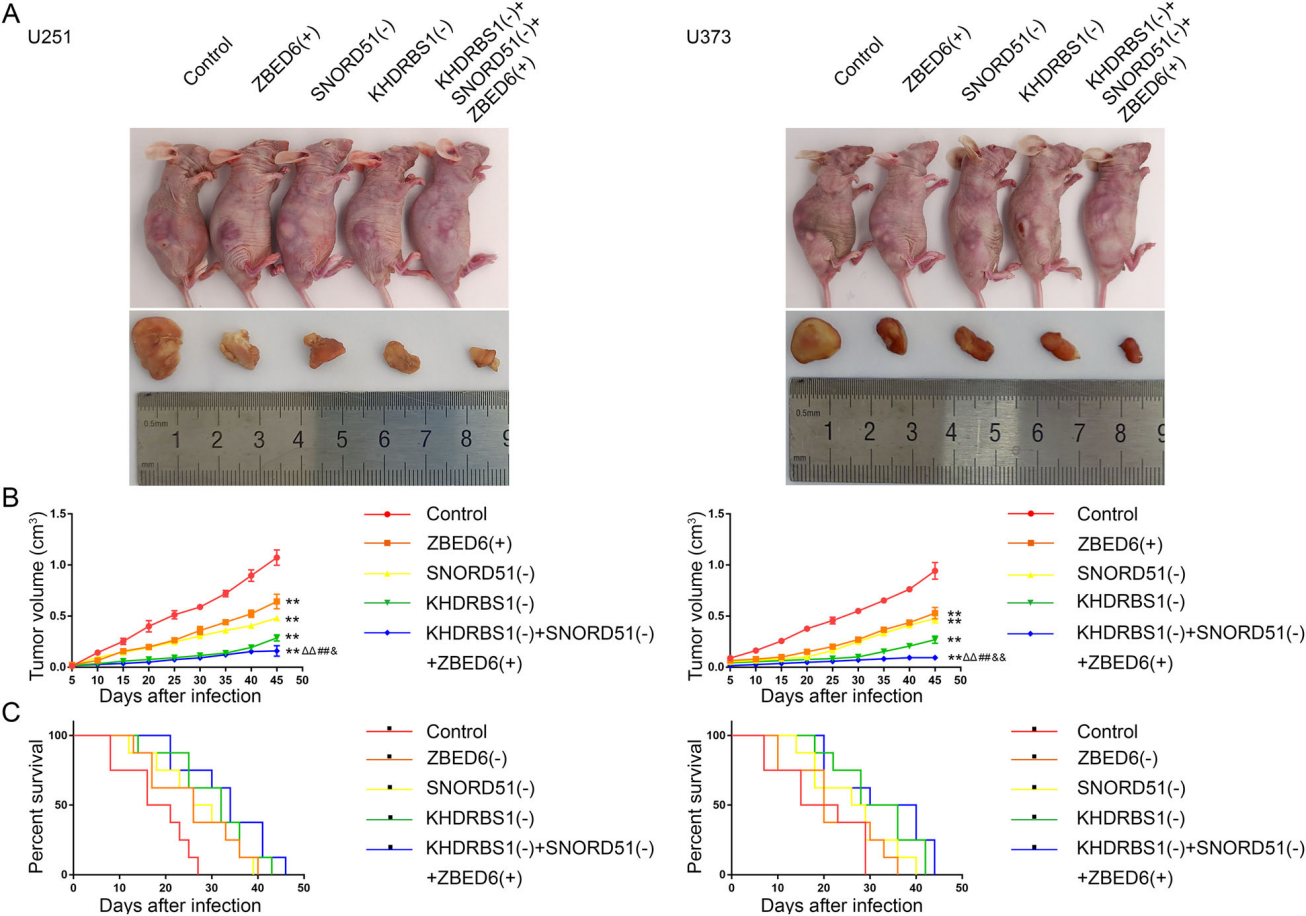
Knockdown of KHDRBS1, SNORD51 and ZBED6 suppressed tumor growth and prolonged survival in nude mice. **A** The nude mice carrying tumors in respective groups are shown. The sample tumors from respective groups are shown. **B** Tumor growth curves are shown. Tumor volume was calculated every 5 days, and tumor was taken at 45 days after injection. Data are presented as the mean ± SD (*n* = 3, each group). ^**^P < 0.01 versus Control group; ^ΔΔ^*P* < 0.01 versus SNORD51(−) group; ^##^*P* < 0.01 versus ZBED6^(+^) group; ^&^*P* < 0.05 versus KHDRBS1(−) group; ^& &^*P* < 0.01 versus KHDRBS1(−) group. **C** Survival curves of nude mice injected into the right striatum are shown (*n* = 8, each group).

In this study, nude mice were divided into five groups to be respectively injected different GBM cells for verifying the roles of KHDRBS1, SNORD51 and ZBED6 in tumor growth. As shown in Fig. 8A, B, nude mice subcutaneously injected with KHDRBS1(−) + SNORD51(−) + ZBED6(+) cells showed a significant reduction in the volume of their tumors compared to nude mice injected with KHDRBS1(−), SNORD51(−) or ZBED6(−) cells alone. GBM cells were implanted into the right striatum of nude mice for survival time analysis (Supplementary Fig. 12A). As shown in Fig. 8C, nude mice injected with KHDRBS1(−) + SNORD51(−) + ZBED6(+) cells showed longer survival time compared to nude mice injected with KHDRBS1(−), SNORD51(−) or ZBED6(−) cells alone.

### Mechanism of the KHDRBS1/SNORD51/ZBED6 pathway to regulate PPP and malignant biological behavior in GBM cells

**Fig. 9:**
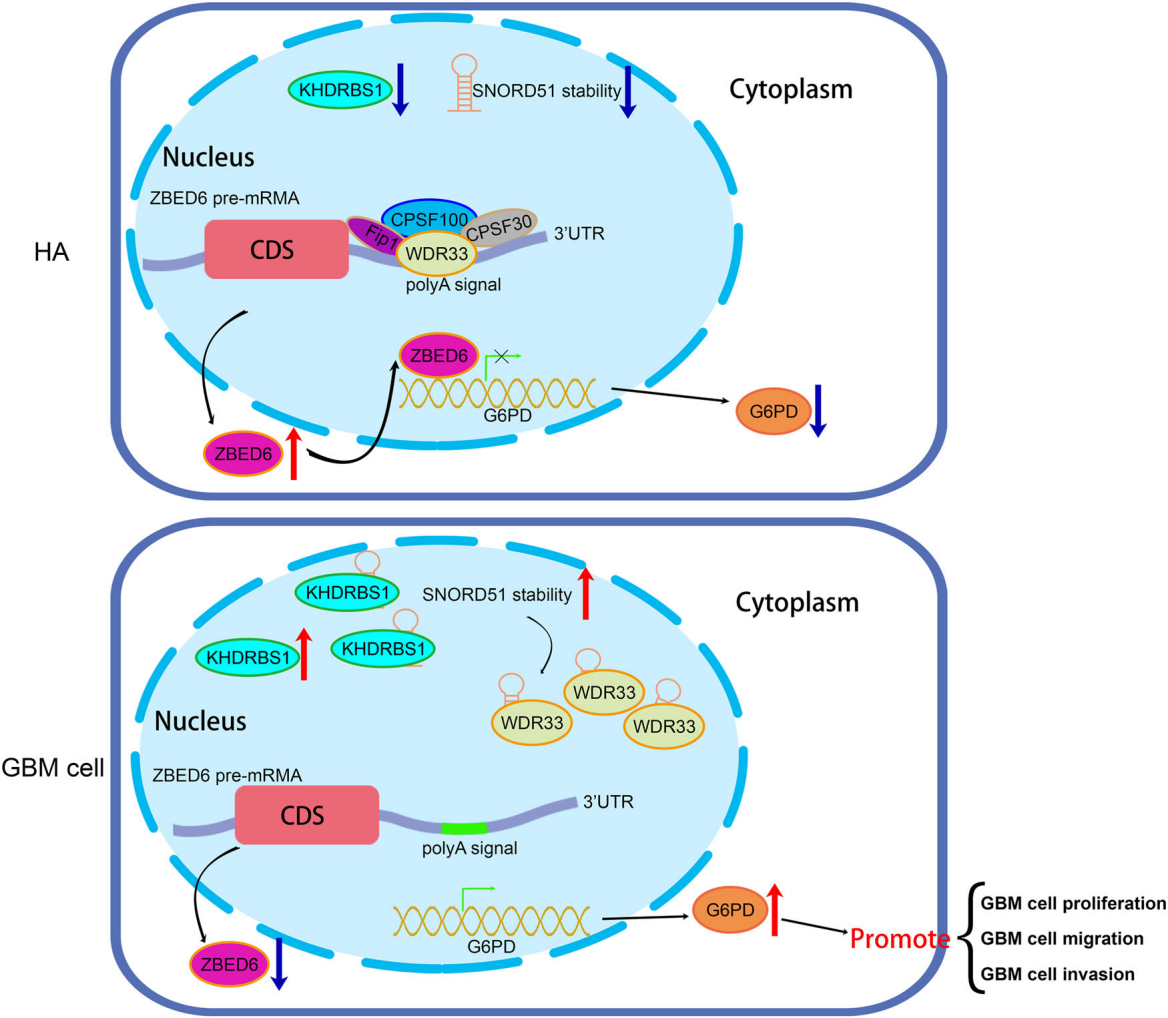
Schematic diagram of KHDRBS1/SNORD51/ZBED6 pathway to regulate the PPP and biological behavior in GBM cells.

Based on the above experimental results, this study demonstrated that the upregulation of KHDRBS1 in GBM cells inhibited the ability of SNORD51 to competitively bind to WDR33 with ZBED6 pre-mRNA through binding to SNORD51 and thus promoted the 3’ end processing of ZBED6 pre-mRNA. In the end, the results of those above processes inhibited the PPP and malignant biological behavior by inhibiting the transcription of G6PD in GBM cells (Fig. 9).

## Discussion

The aberration of metabolism is a prominent character of GBM cells [26, 27]. Metabolism reprogramming abnormally activates the PPP to provide energy and intermediate metabolites for growth and maintain redox balance in tumor cells [28, 29]. Recent studies have found that high levels of ROS increase the risk of tumorigenesis [30]. To adjust with the high levels of ROS, the rapidly proliferated tumor cells enhance the PPP to produce NADPH as antioxidant and satisfy the need for biosynthesis [31, 32]. Accumulating evidence has revealed that the overactivation of the PPP inhibits ferroptosis in clear renal cell carcinoma [33]. The activation of the PPP promotes the malignant biological behavior of renal clear cell carcinoma and pancreatic ductal adenocarcinoma [34, 35]. Nuclear receptor binding SET domain protein 2 (NSD2), which is upregulated in breast cancer, elevates the expression of G6PD to promote the proliferation of breast cancer cells. Fyn phosphorylates 6PGD on Y481 enhancing the enzyme activity of 6PGD to promote the proliferation of GBM cells. Besides, the upregulation of G6PD has been observed in lung cancer and colorectal cancer [36–39]. All these researches indict that the role of PPP in regulating the malignant biological behavior of cancer cells through G6PD and 6PGD [40]. Interestingly, G6PD is upregulated in GBM and the dual inhibition of G6PD and CPT1A significantly inhibits the viability and invasion of GBM [24]. Studies have found that patients with G6PD deficiency have an increased risk of GBM and the upregulation of G6PD in GBM patients does not indicate the poor prognosis [41]. However, the upregulation of G6PD promotes the proliferation of GBM cells [42]. The aberration of the PPP does not have a simple correspondence with the malignant biological behavior of GBM cells. It is urgent for us to pay attention to complex mechanism underneath.

RBPs are involved in RNA splicing, polyadenylation, mRNA stability, mRNA localization and translation, and play a vital role in proliferation, migration, invasion and metastasis [43–45]. KHDRBS1 is upregulated in breast cancer, gastric cancer and prostate cancer which promotes tumorigenesis. KHDRBS1 also regulate metabolic conversion in lung adenocarcinoma and oral squamous cell carcinoma [46–51]. In the present study, we observed that KHDRBS1 was upregulated in GBM and the expression of KHDRBS1 were negatively correlated with survival time of patients. We further detected that KHDRBS1 knockdown significantly inhibited the protein level of G6PD thereby inhibiting the PPP and the proliferation, invasion, migration of GBM cells.

Recent studies demonstrate that the aberrant expression of snoRNAs regulates progression and occurrence of tumors by affecting biological processes. SNORA52 is downregulated in hepatocellular carcinoma (HCC) and has been proved to inhibit its development [52–54]. Many snoRNAs are involved in the processing of mRNAs through interaction with RBPs [55, 56]. KHDRBS1 can regulate the 3’ end processing of pre-mRNA through binding U1 small nuclear ribonucleoprotein (snRNP) [57, 58]. In current study, we found that KHDRBS1 knockdown significantly decreased the expression of SNORD51 in GBM cells. SNORD51 was upregulated in GBM cells and SNORD51 knockdown significantly inhibited the PPP, proliferation, migration and invasion of GBM cells through downregulation of G6PD. We observed that KHDRBS1 and SNORD51 were mainly distributed in the nucleus of GBM cells and confirmed that KHDRBS1 could bind to SNORD51. In addition, this study found that the expression of SNORD51 was significantly decreased and the half-life of SNORD51 was significantly shortened after KHDRBS1 knockdown. SNORD51 overexpression reversed the inhibition on PPP, proliferation, migration and invasion of GBM cells induced by KHDRBS1 knockdown. These results suggest that KHDRBS1 can improve the stability of SNORD51 through binding to it, and then regulate the PPP, proliferation, migration and invasion of GBM cells.

In eukaryotes, the pre-mRNAs obtain stable polyadenylated tails to regulate the length, nuclear transport and translation initiation of mature mRNAs through polyadenylation [59]. Recent studies have found that some mRNAs have the mutual transformation of polyA(+) and polyA(−) to regulate cell cycle, proliferation and apoptosis, which indicates that polyadenylation of mRNAs plays an important role in the development and progression of cancer [60]. Polyadenylation requires the participation of a variety of complexes including cleavage and polyadenylation specificity factor (CPSF), cleavage stimulation factor (CstF), cleavage factor I (CF I) and cleavage factor II (CF II) complexes. WDR33 is a subunit of the CPSF complex, which participates in the 3’ end processing of mRNA by directly binding to the AAUAAA hexamer sequence [61, 62]. Studies have shown that SNORD50A can compete with U-rich sequence existed in cis-element polyadenylation site to bind another subunit of the CPSF complex, factor interacting with polyA polymerase 1 (FIP1) [63]. The genes which contain more U-rich sequences in cis-element polyadenylation sites are upregulated in Hela cells after SNORD50A overexpression, and these genes are related to cell proliferation and apoptosis, suggesting that snoRNAs mediate 3’ processing of mRNAs to regulate cancer development. SNORD51 contains the AAUAAA hexamer sequence. According to the RPISeq, SNORD51 harbored the binding sites for WDR33. In current study, we observed that SNORD51 and WDR33 were mainly distributed in the nucleus of GBM cells and SNORD51 could bind to WDR33. Furthermore, we found SNORD51 markedly upregulated in GBM cells. SNORD51 knockdown inhibited the PPP, proliferation, invasion and migration of GBM cells.

Zinc finger proteins are the largest family of transcription factors in human genome. Recent studies have shown that zinc finger proteins play an important role in the development of cancer [64]. ZBED6 regulates cell metabolism, growth and cycle by inhibiting the expression of IGF2 [15–18]. According to RPISeq, WDR33 might interact with 3’ UTR of ZBED6 mRNA. In this study, we confirmed that the competitive binding WDR33 between the 3’ UTR of ZBED6 mRNA and SNORD51. The mRNA expression of ZBED6 was decreased after SNORD51 overexpression, and WDR33 overexpression reversed the inhibition induced by SNORD51 overexpression. Besides, ZBED6 knockdown restored the inhibitory effects of SNORD51 knockdown in terms of the PPP, proliferation, migration and invasion of GBM cells. All these results indicate that SNORD51 inhibited the expression of ZBED6 by competitively binding to WDR33 with 3’ UTR of ZBED6 mRNA. We also confirmed that ZBED6 inhibited the transcription of G6PD by binding to its promoter region. ZBED6 was downregulated in GBM tissues and cells. ZBED6 overexpression significantly inhibited the PPP, proliferation, migration and invasion of GBM cells. Above results showed that ZBED6 inhibited the PPP by inhibiting the expression of G6PD, thus inhibiting the proliferation, migration and invasion of GBM cells.

Finally, our in vivo study showed that the combination of KHDRBS1 knockdown, SNORD51 knockdown and ZBED6 overexpression significantly reduced the volume of the xenograft GBM tumor and prolonged the survival time of nude mice.

Therefore, KHDRBS1 increased the stability of SNORD51 which promoted the competitive binding ability of SNORD51 to WDR33. Then it inhibited the polyadenylation of 3’ UTR of ZBED6 mRNA and reduced the expression of ZBED6. The downregulated ZBED6 reduced the inhibition of the transcription of G6PD, which promoted the PPP and malignant biological behavior of GBM cells. Our experiments illustrate the role of the KHDRBS1/SNORD51/ZBED6 pathway in regulating PPP of GBM and provide new molecular targets for therapy of GBM.

## Materials and methods

### Bioinformatics analysis

GSE90598, GSE100675 and GSE263588 were obtained from GEO database. The heatmaps, Volcano plot, co-expression correlation heatmap, Sangkey plot, Venn diagrams and ROC curves were used by R (University of Auckland, New Zealand). The GSEA analysis was used by GSEA_4.3.3 software (Broad Institute, Inc., Massachusetts Institute of Technology, and Regents of the University of California, USA).

### Clinical specimens

Clinical samples were obtained from the Department of Neurosurgery in Shengjing Hospital Affiliated China Medical University. For more details, please see Supplementary Methods.

### RNA-sequencing (RNA-seq)

RNA was extracted from SNORD51(−) and SNORD51(−)NC groups. RNA integrity was evaluated using 1% agarose gel electrophoresis, and mRNA-seq libraries were generated. Sequencing libraries were generated using the Hieff NGS^TM^ MaxUp Dual-mode mRNA Library Prep Kit for Illumina (Illumina, CA, USA). The gene expression values of the transcripts were analyzed by Sangon Biotech (Sangon Biotech, Shanghai, China).

### Cell culture

Cell culture was performed as previously described [65]. For more details, please see Supplementary Methods.

### RNA extraction and quantitative real-time PCR

RNA extraction and quantitative real-time PCR were performed as previously described [65].The primers were provided in Supplementary Table S3. β-actin and U6 were used as internal references. The relative quantification of above was calculated to the 2^−ΔΔCt^ value. For more details, please see Supplementary Methods.

### Western blot

Western blot was performed as previously described [65]. The primary antibodies were shown in Supplementary Table S5. For more details, please see Supplementary Methods.

### FISH

FISH was performed as previously described [65]. The SNORD51 probe was shown in Supplementary Table S3. For more details, please see Supplementary Methods.

### Cell transfection

Cell transfection was performed as previously described [65]. For details, see Supplementary Table S6 and Supplementary Fig. 9. For more details of experiment, please see Supplementary Methods.

### IF

IF was performed as previously described [65]. Antibodies used were provided in Supplementary Table S5. For more details of experiment, please see Supplemental Methods.

### Relative ROS production assay

Reactive Oxygen Species Assay Kit (Beyotime, Jiangsu, China) was used to detect the relative ROS level of cells according to the manufacturer’s instructions.

### NADP + /NADPH assay

NADP + /NADPH Assay Kit with WST-8 (Beyotime, Jiangsu, China) was used to detect the ratio of NADP + /NADPH of the cells.

### Cell proliferation assay

Cell proliferation assay was performed as previously described [65]. For more details of experiment, please see Supplementary Methods.

### Cell migration assay

Cells were inoculated onto 6-well plates (Corning, NJ, USA) at appropriate amount and cultured under HoloMonitor M4 system for observation.

### Cell invasion assay

Cell invasion assay was performed as previously described [65]. For more details of experiment, please see Supplementary Methods.

### RNA stability measurement

RNA stability measurement was performed as previously described [66]. For more details of experiment, please see Supplementary Methods.

### RIP Assay

RIP assay was performed as previously described [65]. For more details of experiment, please see Supplementary Methods.

### RNA-Pull down assay

RNA-Pull down assay was performed as previously described [65]. For more details of experiment, please see Supplementary Methods.

### In Vitro transcription for probes preparation

The non-biotin-labeled T7 in vitro transcription kit and biotin-labeled T7 in vitro transcription kit (RIOBIO, Guangzhou, China) were used to transcriptionally amplify biotin-labeled or non-biotin-labeled RNA probes in vitro by pairs of specific primers containing T7 promoter sequence. Then, the products were verified as target sequence by Sanger sequencing. Details were shown in Supplementary Materials.

### Nuclear protein extraction

By using Nuclear and Cytoplasmic Protein Extraction Kit (Beyotime, Jiangsu, China), cells collected were mixed with cytoplasmic protein extraction reagent A supplemented with PMSF. Cells were lysed on ice for 10-15 min and then cytoplasmic protein extraction reagent B was added and lysed on ice for 1 min, followed by centrifugation at 12,000-16,000 g for 5 min at 4 °C. Discard the supernatant, the remains were lysed on ice by adding nuclear protein extraction reagent supplemented with PMSF, followed by high-speed shaking for 15-20 s at 1-2 min intervals. The whole process lasted for 30 min. In the end, by centrifugation at 12,000-16,000 g for 10 min at 4 °C, the supernatant was extracted as the nuclear protein extraction.

### Competitive electrophoretic mobility shift assay

According to the manufacturer’s instructions, the EMSA assay was carried out by using chemiluminescent EMSA kit (Beyotime, Jiangsu, China). The nuclear extractions were divided into WDR33-immunodepleted group and negative control group. These groups were incubated with the biotin-labeled 3’UTR of ZBED6 probe respectively. The non-biotin-labeled SNORD51 probe acted as competitor, which can weaken shift band by competitively binding to WDR33. After 20 min incubation, the mixture was subjected to 5% polyacrylamide gel electrophoresis and the band was transferred to a Nylon membrane (Beyotime, Jiangsu, China). Followed by UV crosslinking, the band was visualized by this kit and scanned by ChemImager 5500 V2.03 software.

### Rapid amplification of cDNA end and Sanger sequencing

RACE and Sanger sequencing were performed as previously described [66]. For more details, please see Supplementary Methods.

### Chromatin immunoprecipitation assay

ChIP assay was performed as previously described [66]. For more details, please see supplemental methods.

### Luciferase reporter assay

Human full-length ZBED6 gene was constructed into pEX3 vector (GenePharma, Shanghai, China). The reporter vector construction was carried out as previously described [65]. Details were shown in Supplementary Materials.

### Tumor xenograft in nude mice

Tumor xenograft models in nude mice were constructed as previously descrbied [66]. Details were shown in Supplemental Methods.

### H&E staining

Place the slides at 60 °C for 90 min. Transfer the slides into staining jar with xylene for 5 min.Successively transfer the slides into staining jars with xylene for 5 min, 100% ethanol for 5 min, 90% ethanol for 2 min, 70% ethanol for 2 min. Transfer the slides to a staining jar with distilled water for 2 min and incubate the slides with hematoxylin solution (Solarbio, Beijing, China) in a staining jar for 10 min to stain the nuclei. Transfer the slides to a staining jar with running water (tap water is fine) till the water is clear. Transfer the slides to a staining jar with Eosin solution (Solarbio, Beijing, China) for 3 min. Successively transfer the slides into staining jars with 70% ethanol for 20 sec, 90% ethanol for 20 sec, 100% ethanol for 1 min and xylene for 3 min. Take out slides from xylene and place the slides in a fume hood till the slides are dry. Mount the slides with xylene-based mounting media and cover with cover slides. Clips are used to press the slides to squeeze bubbles. Store the slides at room temperature. Hematoxylin and Eosin-stained images were captured by Aperio Versa 8 (Leica, German).

### Statistical analysis

The experimental data were collected and presented as mean ± SD. Student’s t-test or one-way ANOVA or two-way ANOVA was used in the statistical analysis by the GraphPad Prism v7 (GraphPad, CA, USA). Differences between groups were regarded as significant when *P* < 0.05.

## Supplementary information

Supplementary figures and tables

Supplemental methods

Original Data File

## Data Availability

All data and materials analyzed or used to support the findings in this study are available from the corresponding author upon a reasonable request.
