# Supplementary material for: KHDRBS1 regulates the pentose phosphate pathway and malignancy of GBM through SNORD51-mediated polyadenylation of ZBED6 pre-mRNA
Source: Cell Death Dis. 2024 Nov 8;15(11):802. doi: 10.1038/s41419-024-07163-x (PMC11549417; doi:10.1038/s41419-024-07163-x)
Supplement: Supplementary file 1 — Supplementary figures and tables [file 41419_2024_7163_MOESM1_ESM.docx]

**Supplementary Figure**

**Supplementary figure 1**

**
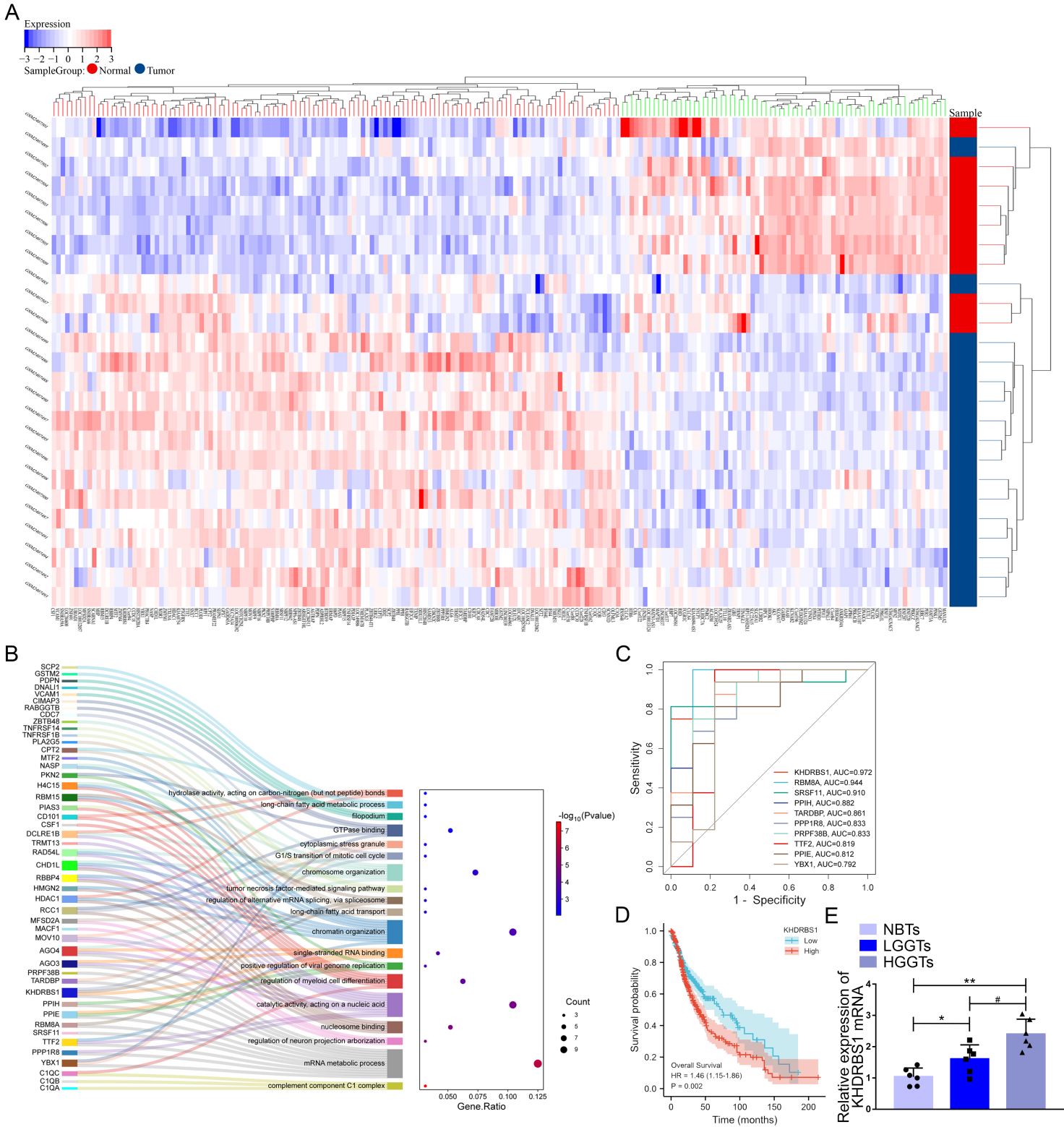
**

**Supplementary figure 1. KHDRBS1 was selected as a research candidate.**

(A). Based on the GEO dataset GSE90598, we selected 200 differentially expressed protein-coding genes in GBM cells. (B). Sangkey plot showed the results of Gene Ontology enrichment analysis of top 50 upregulated protein coding genes according to LogFC. (C). ROC curves of top 10 genes related to mRNA metabolic process accroding to AUC based on GEO dataset GSE90598. (D). Effect of KHDRBS1 expression level on glioma patient survival time from TCGA database. (E). The mRNA expression levels of KHDRBS1 in NBTs, LGGTs and HGGTs. Data are presented as the mean ± SD (n=6, each group). *^*^P* < 0.05 versus NBTs group; *^**^P* < 0.01 versus NBTs group; ^#^*P* < 0.05 versus LGGTs group.

**Supplementary figure 2**


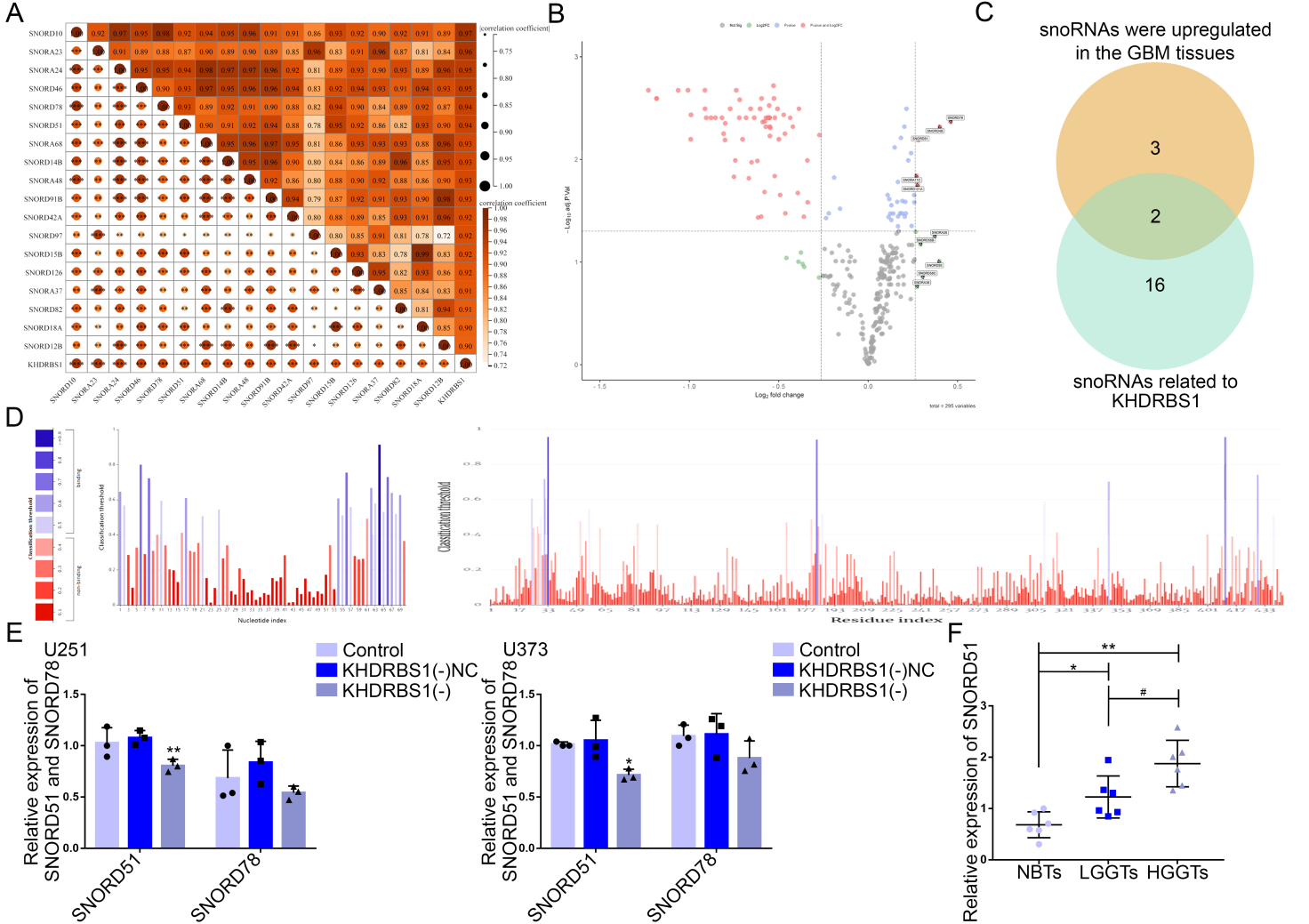


**Supplementary figure 2. SNORD51 was selected as a research candidate and was upregulated in GBM tissues.**

(A). According to the GEO dataset GSE100675, the co-expression correlation heatmap of KHDRBS1 and 18 snoRNAs selected by ranking their correlation coefficients. (B). Volcano plot of differentially expressed snoRNAs in GSE100675. (C). Venn diagram of upregulated snoRNAs and snoRNAs related to KHDRBS1 in GBM tissues. (D). According to RNA Interactome Database (RNAInter), KHDRBS1 may interact with SNORD51. (E). Effects of KHDRBS1 knockdown on the expression of SNORD51 and SNORD78 in GBM cells. Data are presented as the mean ± SD (n=3, each group). ^*^*P* < 0.05 versus KHDRBS1(-)NC group; ^**^*P* < 0.01 versus KHDRBS1(-)NC group. (F). The expression of SNORD51 was detected in NBTs, LGGTs and HGGTs. Data are presented as the mean ± SD (n=6, each group). ^*^*P* < 0.05 versus NBTs group; ^**^*P* < 0.01 versus NBTs group; ^#^*P* < 0.05 versus LGGTs group.

**Supplementary figure 3**

**
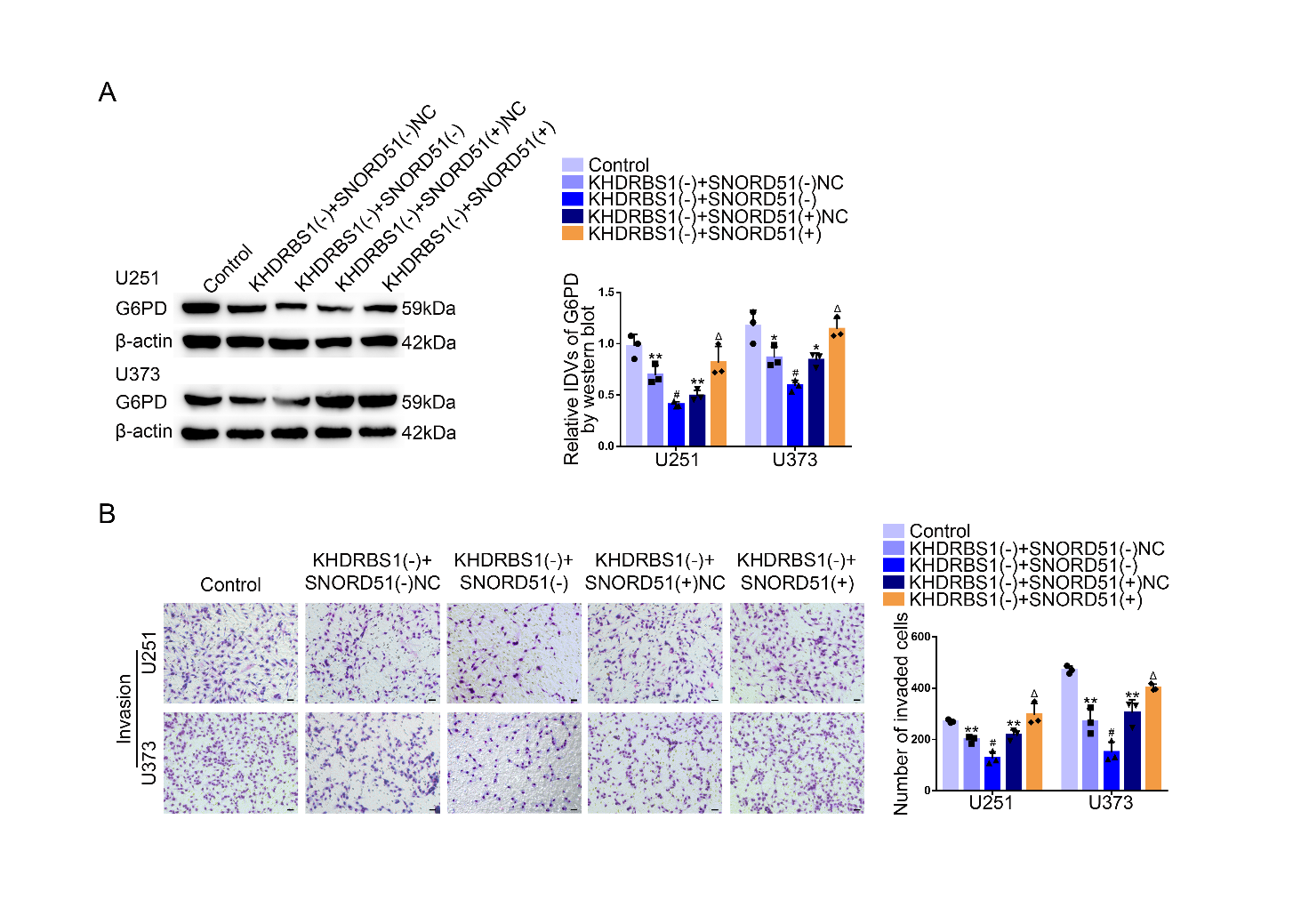
**

**Supplementary figure 3. Combined effects of KHDRBS1 and SNORD51 on expression of G6PD and invasion of GBM cells.**

(A). The reverse effect of SNORD51 on KHDRBS1 in term of G6PD expression level. Data are presented as the mean ± SD (n=3, each group). ^*^*P <* 0.05 versus Control group; ^**^*P <* 0.01 versus Control group; ^#^*P* < 0.01 vs KHDRBS1(-) + SNORD51(-)NC group; ^Δ^*P <* 0.05 versus KHDRBS1(-)+SNORD51(+)NC group. (B). The reverse effect of SNORD51 on KHDRBS1 in term of invasion of GBM cells. Data are presented as the mean ± SD (n=3, each group). ^**^*P <* 0.01 versus Control group; ^#^*P* < 0.01 vs KHDRBS1(-) + SNORD51(-)NC group; ^Δ^*P <* 0.05 versus KHDRBS1(-) + SNORD51(+)NC group.

**Supplementary figure 4**

**
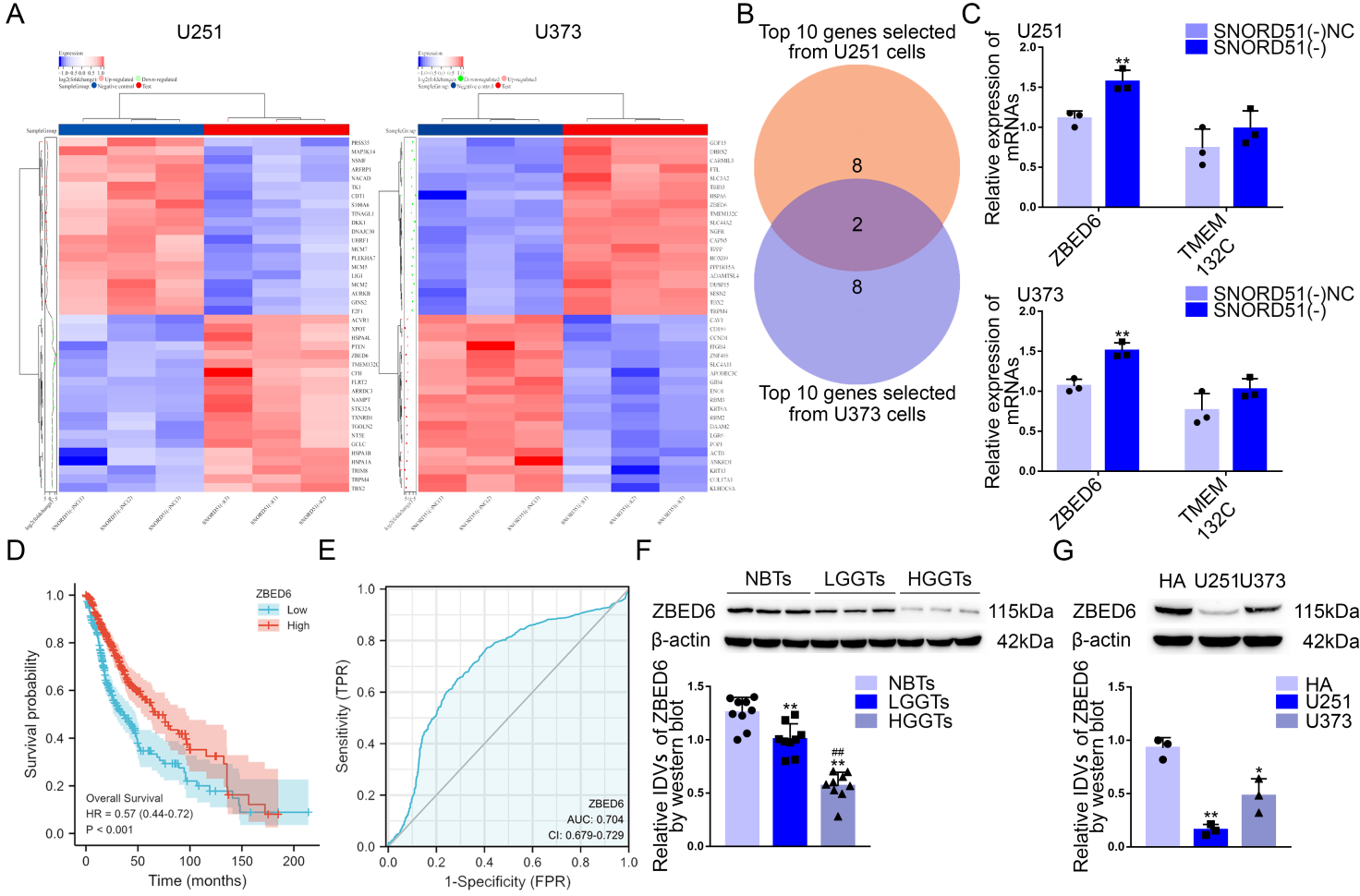
**

**Supplementary figure 4. ZBED6 was selected as a research candidate and was downregulated in GBM tissues and cells.**

(A). The top 40 protein-coding genes that were differentially expressed after knockdown of SNORD51 in GBM cells were screened by mRNA sequencing based on LogFC. (B). Venn diagram was to screen out ZBED6 and TMEM132C that were both upregulated after knockdown of SNORD51 in U251 and U373 cells. (C). Effects of SNORD51 knockdown on the expression of ZBED6 and TMEM132C in GBM cells. Data are presented as the mean ± SD (n=3, each group). ^*^*P* < 0.05 versus SNORD51(-)NC group; ^**^*P* < 0.01 versus SNORD51(-)NC group. (D). Effect of ZBED6 expression level on glioma patient survival time from TCGA database. (E). ROC curve of ZBED6 mRNA expression in glioma cohort of TCGA database. (F). The protein expression level of ZBED6 was detected in NBTs, LGGTs and HGGTs. Data are presented as the mean ± SD (n=9, each group). ^*^^*^*P* < 0.01 versus NBTs group; ^##^*P* < 0.01 versus LGGTs group. (G). The protein expression level of ZBED6 was detected in human astrocytes (HA) and GBM lines (U251 and U373) by western blot. Data are presented as the mean ± SD (n=3, each group). ^*^*P* < 0.05 versus HA group; ^*^^*^*P* < 0.01 versus HA group.

**Supplementary figure 5**

**
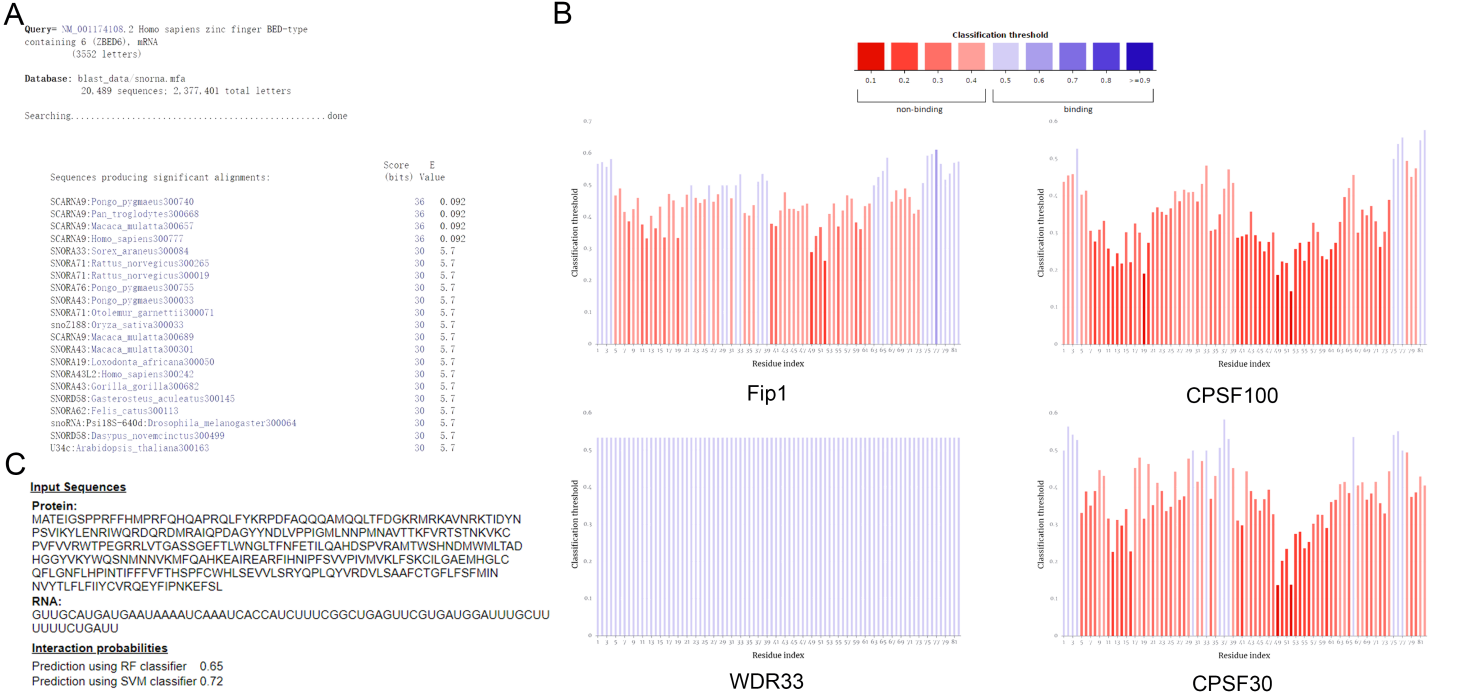
**

**Supplementary figure 5. The prediction of SNORD51 and WDR33.**

(A). Based on snoRNA Orthological Gene Database (SNOOPY), SNORD51 did not have complementary sequence of ZBED6 pre-mRNA. (B). Based on RNAInter, the results suggested that WDR33 may be more likely to combine with SNORD51. (C). Based on RNA-Protein interaction prediction (RPISeq), WDR33 may bind to SNORD51.

**Supplementary figure 6**

**
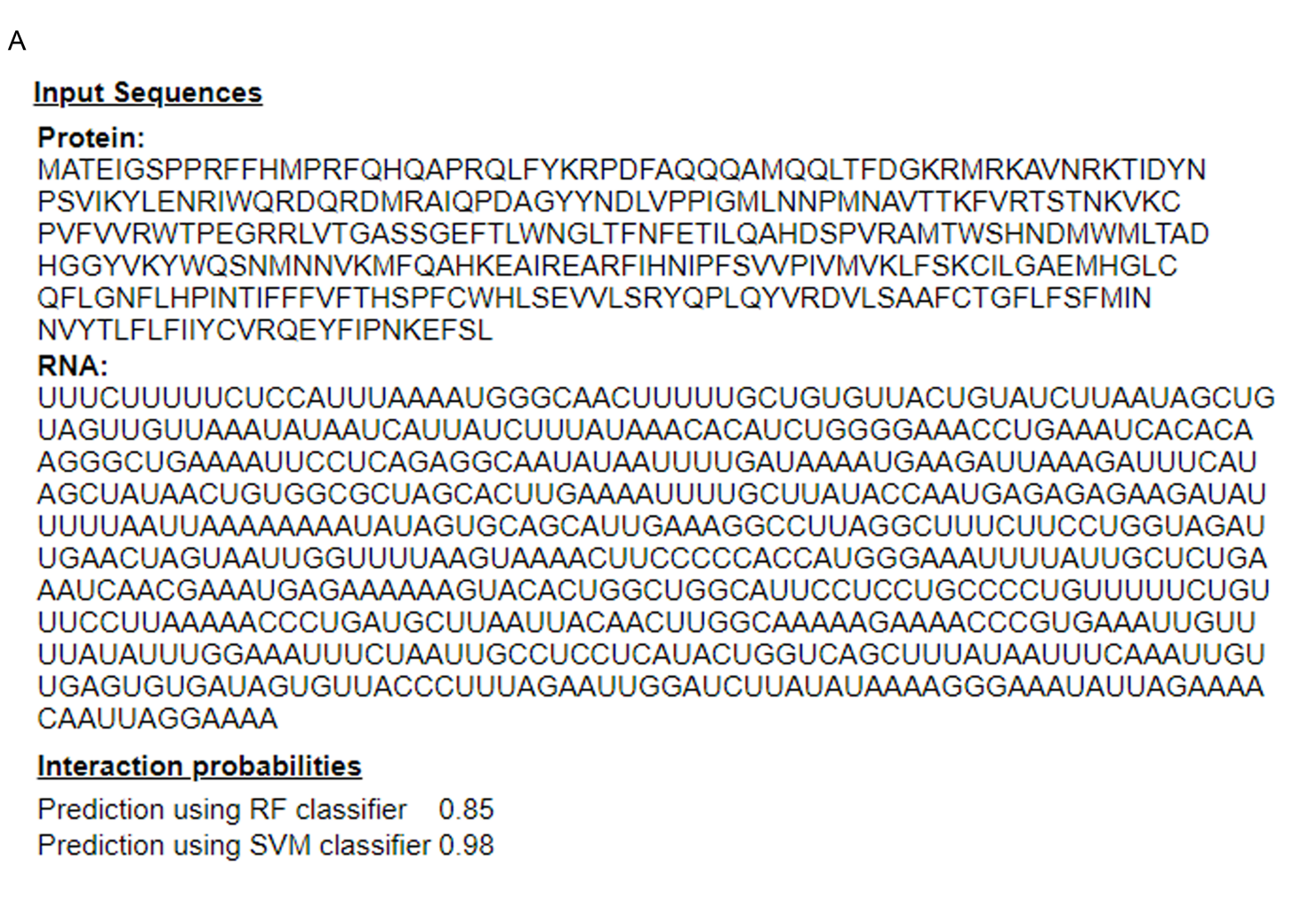
**

**Supplementary figure 6. The prediction of 3’UTR of ZBED6 mRNA and WDR33.**

(A). According to RNA-Protein Interaction Prediction (RPISeq), the 3’UTR of ZBED6 mRNA may contain putative binding sites of WDR33.

**Supplementary figure 7**

**
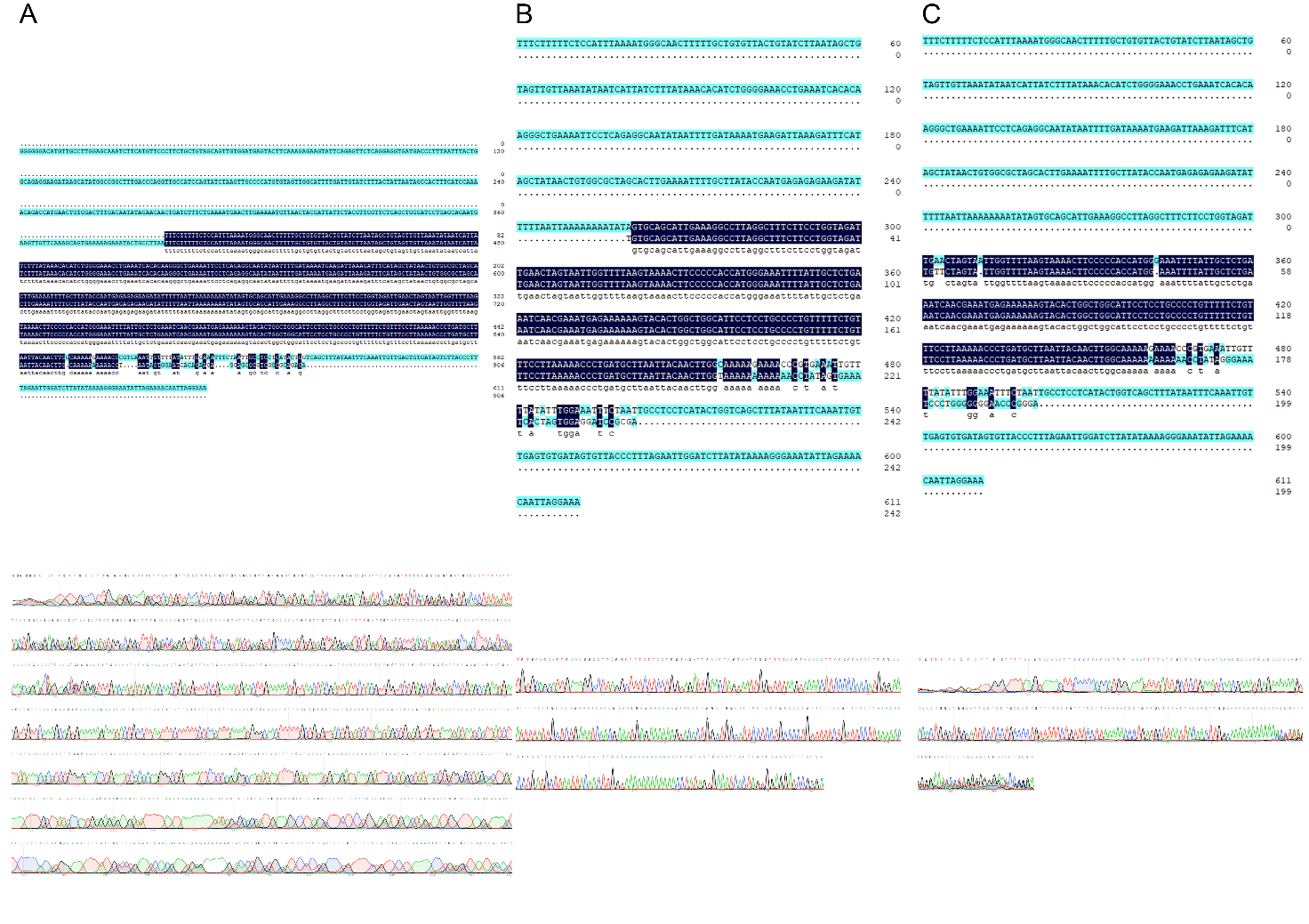
**

**Supplementary figure 7. The results of Sanger sequencing and sequence alignment.**

(A)-(C). The sequences of 3’RACE of ZBED6 mRNA was detected by Sanger sequencing. The results of sequence alignment between original 3’UTR of ZBED6 pre-mRNA and polyadenylated 3’end sequence of ZBED6 mRNA obtained from 3’RACE by different gene specific primers.

**Supplementary figure 8**


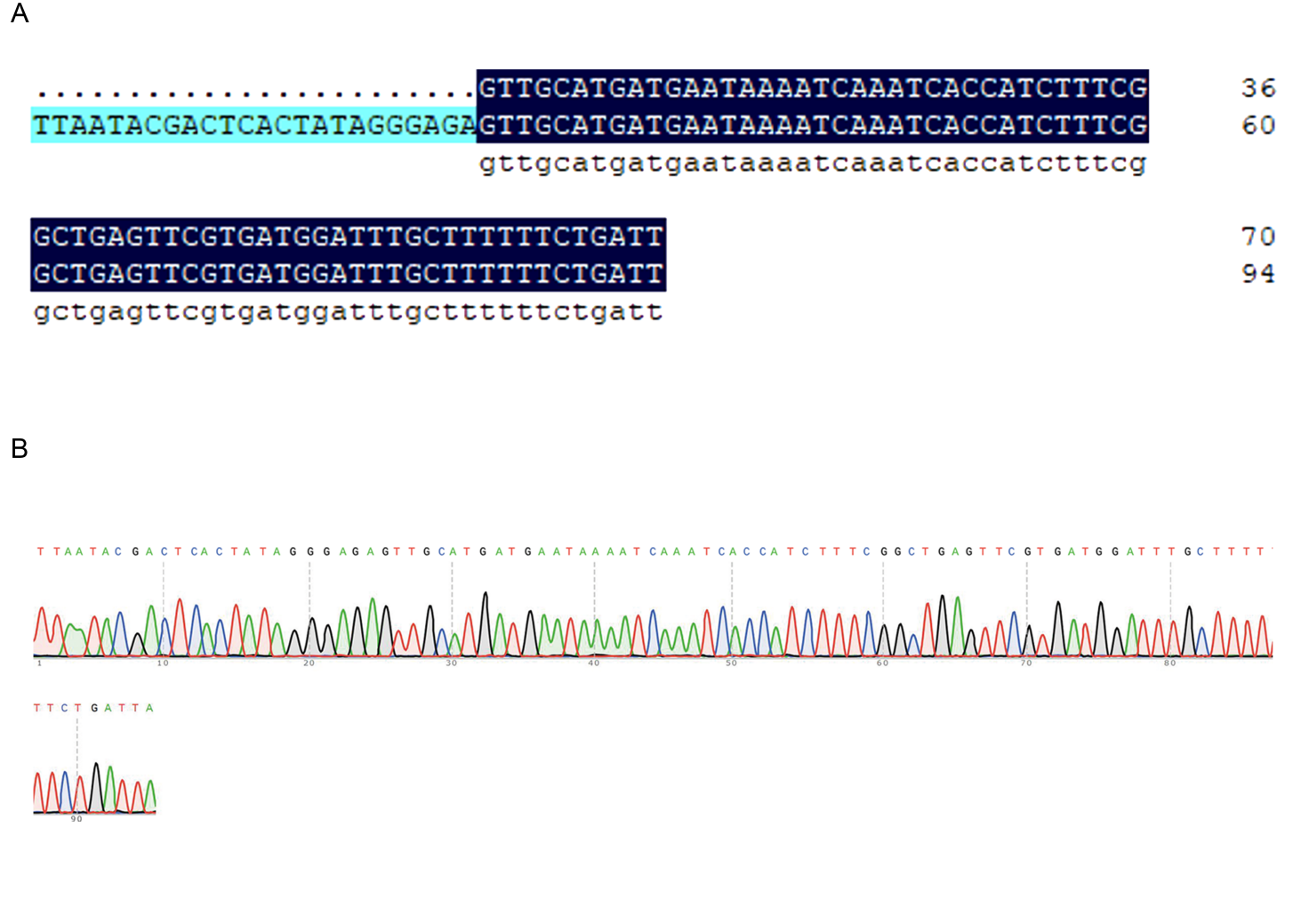


**Supplementary figure 8. The preparation for SNORD51 probe.**

(A). The result of sequence alignment between original SNORD51 and SNORD51 probe for in vitro transcription. (B). The sequence of SNORD51 probe was detected by Sanger sequencing.

**Supplementary figure 9**


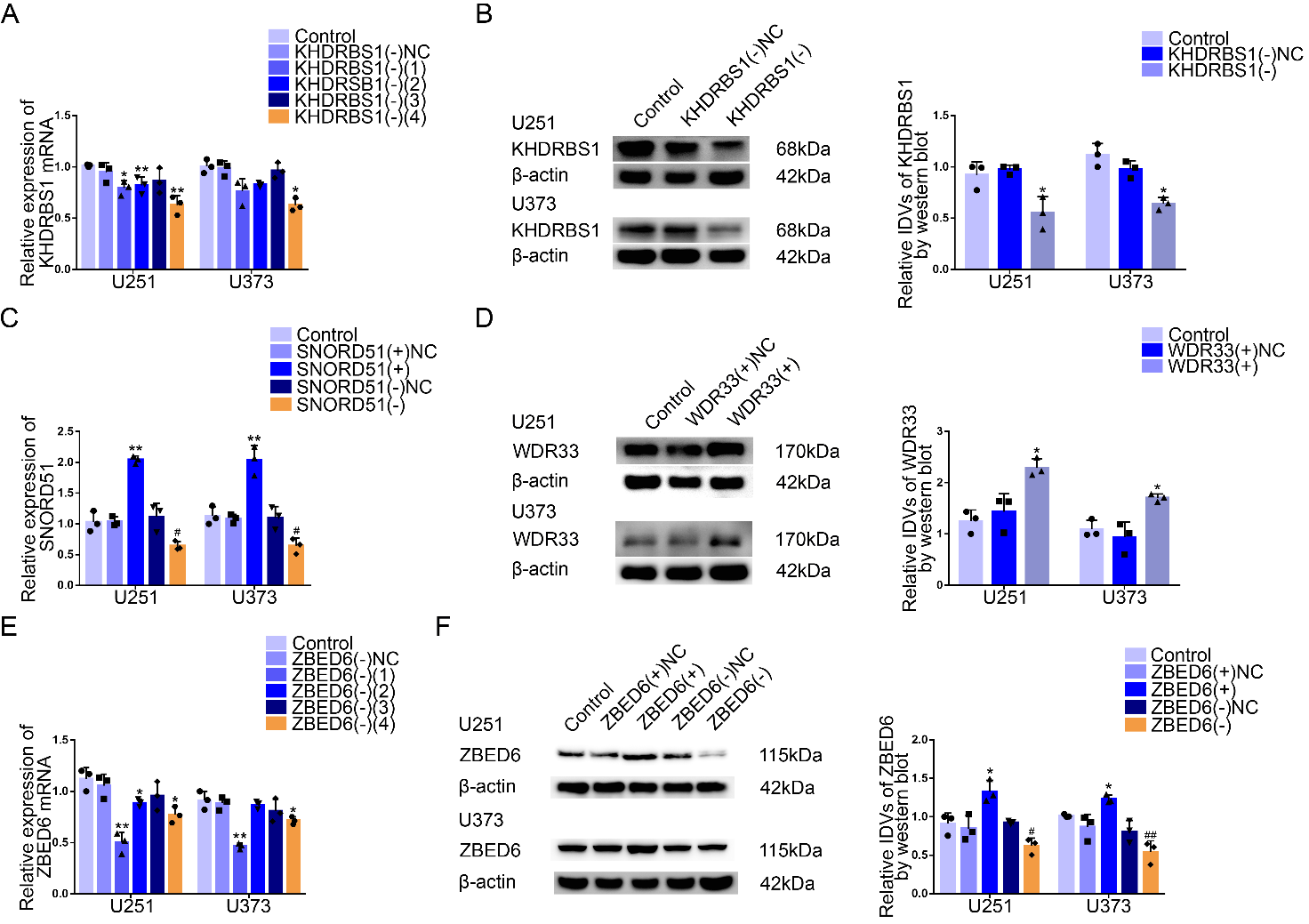


**Supplementary figure 9. The transfection efficiency of KHDRBS1, SNORD51 WDR33 and ZBED6 in GBM cells.**

(A). Efficiency of stable KHDRBS1(-) at four different sites were detected by RT-qPCR. Data are presented as the mean ± SD (n=3, each group). ^*^*P <* 0.05 versus KHDRBS1(-)NC group. ^**^*P <* 0.01 versus KHDRBS1(-)NC group. (B). Efficiency of stable KHDRBS1(-) were detected by western blot. Data are presented as the mean ± SD (n=3, each group). ^*^*P <* 0.05 versus KHDRBS1(-)NC group. (C). Efficiency of stable SNORD51(-) and SNORD51(+) were detected by RT-qRCR. Data are presented as the mean ± SD (n=3, each group). ^**^*P <* 0.01 versus KHDRBS1(-)NC group. ^#^*P <* 0.05 versus KHDRBS1(+)-NC group. (D). Efficiency of stable WDR33(+) were detected by western blot. Data are presented as the mean ± SD (n=3, each group). ^*^*P <* 0.05 versus WDR33(+)NC group.(E). Efficiency of stable ZBED6(-) at four different sites were detected by RT-qPCR. Data are presented as the mean ± SD (n=3, each group). ^*^*P <* 0.05 versus ZBED6(-)NC group. ^**^*P <* 0.01 versus ZBED6(-)NC group. (F). Efficiency of stable ZBED6(-) and ZBED6(+) were detected by western blot. Data are presented as the mean ± SD (n=3, each group). ^*^*P <* 0.05 versus ZBED6(-)NC group. ^#^*P <* 0.05 versus ZBED6(+)NC group. ^##^*P <* 0.05 versus ZBED6(+)NC group.

**Supplementary figure 10**


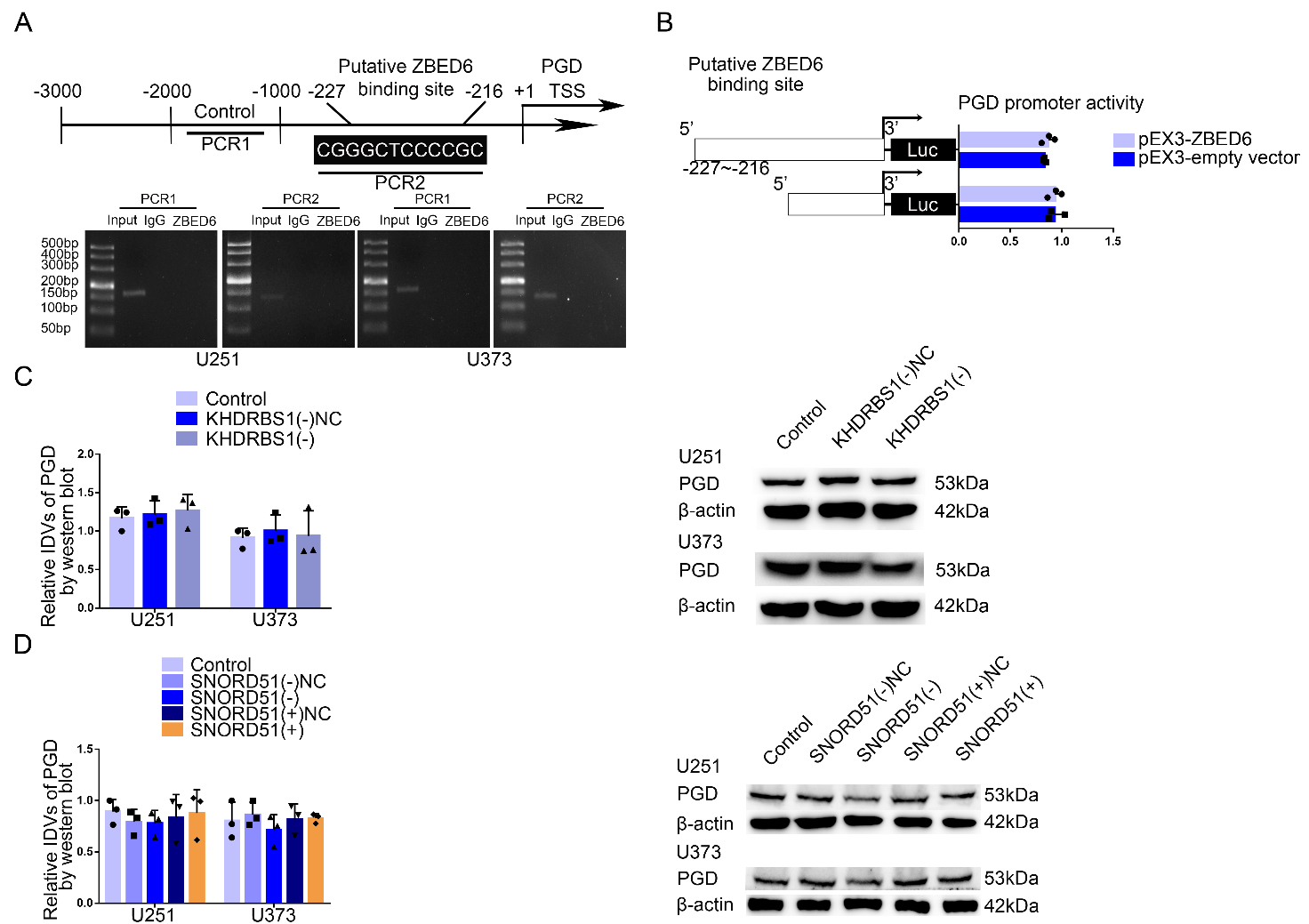


**Supplementary figure 10. Regulatory effect of ZBED6 on transcription of PGD and effects of KHDRBS1 and SNORD51 on the expression of PGD.**

(A). Putative ZBED6 binding sites on the promoter region of PGD were determined by ChIP assays in U251 and U373 cells. Transcription start sites (TSSs) were designated as +1. PCR was conducted with the resulting precipitated DNA. (B). Schematic depiction of different reporter vectors and relative luciferase activity of G6PD. Data are presented as the mean ± SD (n=3, each group). (C). KHDRBS1 knockdown had no effect on the expression of PGD in U251 and U373 cells. Data are presented as the mean ± SD (n=3, each group). (D). The altered expression of SNORD51 had no effect on the expression of PGD in U251 and U373 cells. Data are presented as the mean ± SD (n=3, each group).

**Supplementary figure 11**

**
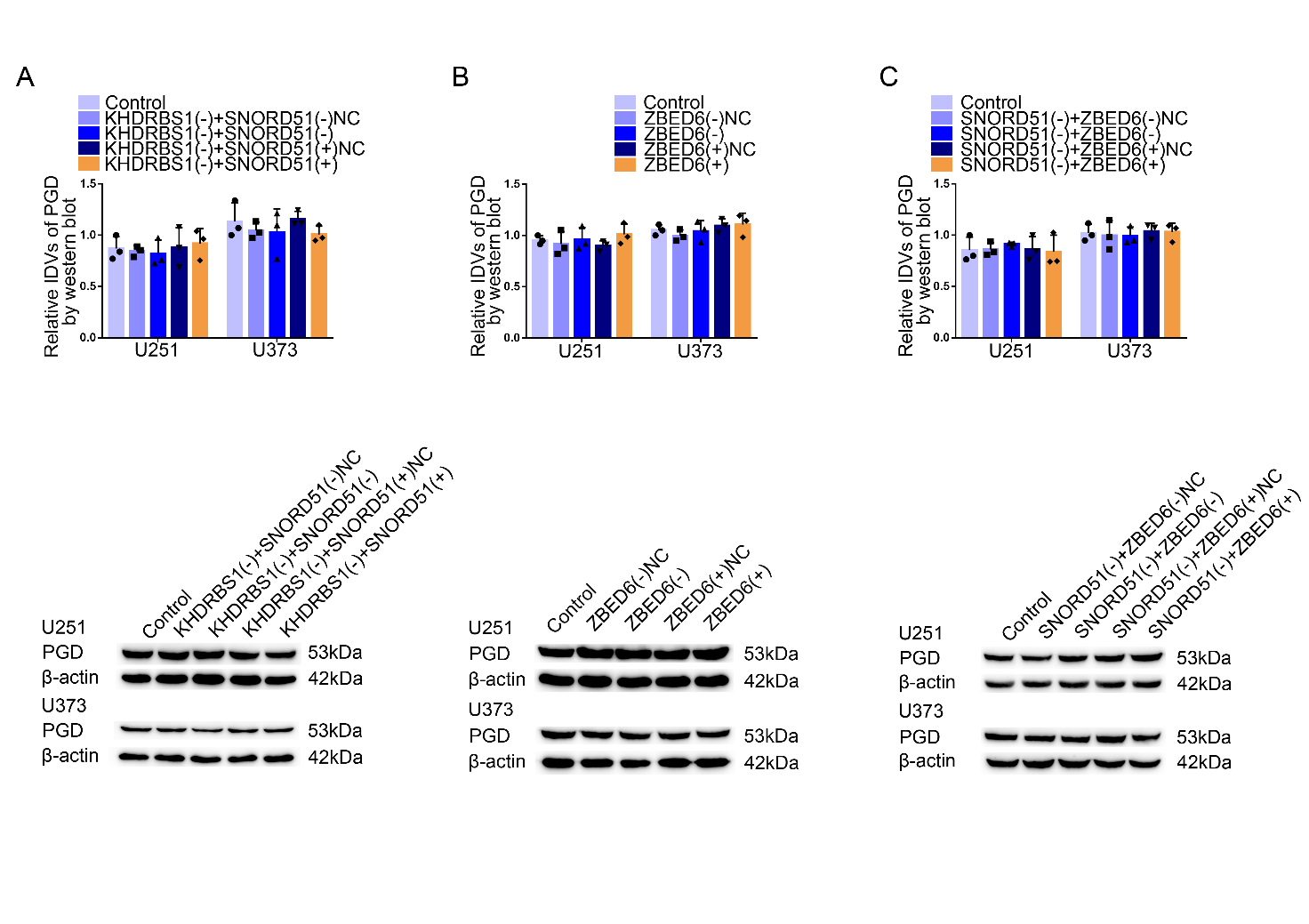
**

**Supplementary figure 11. Combined effects of KHDRBS1 and SNORD51, effect of ZBED6 and combined effects of SNORD51 and ZBED6 on expression of PGD in GBM cells.**

(A). Combination of altered expression of KHDRBS1 and SNORD51 had no effect on the expression of PGD in GBM cells. Data are presented as the mean ± SD (n=3, each group). (B). Altered expression of ZBED6 had no effect on the expression of PGD in GBM cells. Data are presented as the mean ± SD (n=3, each group). (C). Combination of altered expression of SNORD51 and ZBED6 had no effect on the expression of 6PGD in GBM cells. Data are presented as the mean ± SD (n=3, each group).

**Supplementary figure 12**

**
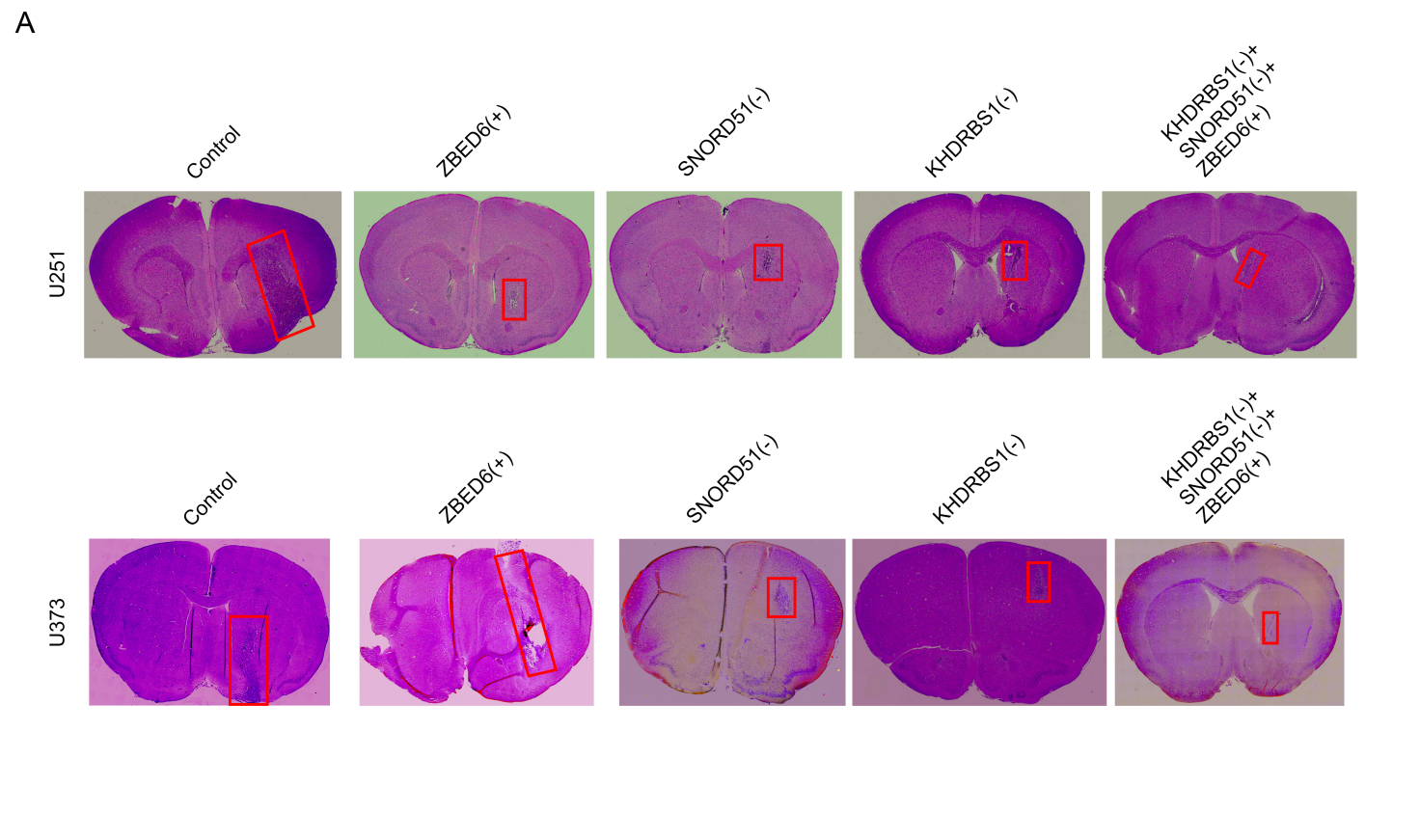
**

**Supplementary figure 12. Knockdown of KHDRBS1、SNORD51 and ZBED6 suppressed tumor growth and prolonged survival in nude mice.**

(A). Representative H&E staining images of brain cross-section of nude mice. Red boxes indicted the area of the GBM tumors.

**Supplementary Table**

**Supplementary table 1**

**Clinical and Gene Expression Data of KHDRBS1 from TCGA Database**

| **Characteristic** | **levels** | **Low expression of KHDRBS1** | **High expression of KHDRBS1** | **p** |
| --- | --- | --- | --- | --- |
| n |  | 348 | 348 |  |
| WHO grade, n (%) | G2 | 133 (20.9%) | 91 (14.3%) | < 0.001 |
|  | G3 | 113 (17.8%) | 130 (20.5%) |  |
|  | G4 | 66 (10.4%) | 102 (16.1%) |  |
| IDH status, n (%) | WT | 98 (14.3%) | 148 (21.6%) | < 0.001 |
|  | Mut | 245 (35.7%) | 195 (28.4%) |  |
| 1p/19q codeletion, n (%) | codel | 140 (20.3%) | 31 (4.5%) | < 0.001 |
|  | non-codel | 205 (29.8%) | 313 (45.4%) |  |
| Primary therapy outcome, n (%) | PD | 42 (9.1%) | 70 (15.2%) | 0.005 |
|  | SD | 78 (16.9%) | 69 (14.9%) |  |
|  | PR | 39 (8.4%) | 25 (5.4%) |  |
|  | CR | 79 (17.1%) | 60 (13%) |  |
| Gender, n (%) | Female | 149 (21.4%) | 149 (21.4%) | 1.000 |
|  | Male | 199 (28.6%) | 199 (28.6%) |  |
| Race, n (%) | Asian | 3 (0.4%) | 10 (1.5%) | 0.133 |
|  | Black or African American | 18 (2.6%) | 15 (2.2%) |  |
|  | White | 319 (46.7%) | 318 (46.6%) |  |
| Age, n (%) | <=60 | 282 (40.5%) | 271 (38.9%) | 0.348 |
|  | >60 | 66 (9.5%) | 77 (11.1%) |  |
| Age, meidan (IQR) |  | 45 (35, 57.25) | 46 (34, 59.25) | 0.863 |

**Supplementary table 2**

**Clinical and Gene Expression Data of ZBED6 from TCGA Database**

| **Characteristic** | **levels** | **Low expression of ZBED6** | **High expression of ZBED6** | **p** |
| --- | --- | --- | --- | --- |
| n |  | 348 | 348 |  |
| WHO grade, n (%) | G2 | 95 (15%) | 129 (20.3%) | < 0.001 |
|  | G3 | 84 (13.2%) | 159 (25%) |  |
|  | G4 | 143 (22.5%) | 25 (3.9%) |  |
| IDH status, n (%) | WT | 160 (23.3%) | 86 (12.5%) | < 0.001 |
|  | Mut | 180 (26.2%) | 260 (37.9%) |  |
| 1p/19q codeletion, n (%) | codel | 53 (7.7%) | 118 (17.1%) | < 0.001 |
|  | non-codel | 289 (41.9%) | 229 (33.2%) |  |
| Primary therapy outcome, n (%) | PD | 35 (7.6%) | 77 (16.7%) | 0.182 |
|  | SD | 54 (11.7%) | 93 (20.1%) |  |
|  | PR | 25 (5.4%) | 39 (8.4%) |  |
|  | CR | 62 (13.4%) | 77 (16.7%) |  |
| Gender, n (%) | Female | 144 (20.7%) | 154 (22.1%) | 0.491 |
|  | Male | 204 (29.3%) | 194 (27.9%) |  |
| Race, n (%) | Asian | 7 (1%) | 6 (0.9%) | 0.936 |
|  | Black or African American | 17 (2.5%) | 16 (2.3%) |  |
|  | White | 316 (46.3%) | 321 (47%) |  |
| Age, n (%) | <=60 | 257 (36.9%) | 296 (42.5%) | < 0.001 |
|  | >60 | 91 (13.1%) | 52 (7.5%) |  |
| Age, meidan (IQR) |  | 49 (36, 62) | 41 (32, 55) | < 0.001 |

**Supplementary table 3. Primers used for qRT-PCR and the probe for FISH assays**

| **Primer or Probe** | **Gene** | **Sequence (5’->3’)** |
| --- | --- | --- |
| Primer | KHDRBS1 | F: GATGGAGCCAGAGAACAAGTAC |
|  |  | R: TGAATCTTCTCAATTTCTGCCG |
|  | β-actin | F: GTGCTATCCCTGTACGCCTC |
|  |  | R: AATGCCAGGGTACATGGTGG |
|  | ZBED6 | F: CTTGGTAGAGCCAGTGGAGTTGTG |
|  |  | R: ACCTGATTCAATCCTGCGGTCTTC |
|  | ZIC3 | F: ACAACCACGTCTGCTACTGG |
|  |  | R: GAGGAAGTCCAGGGTTGTGG |
|  | RBM15 | F: GCCTCTGCCCTTGACTCATT |
|  |  | R: GTCCGGTGCTTCCTATCTCG |
| Probe | SNORD51 | GCCGAAAGATGGTGATTTGAGCAAATCCATCACGAACTCA |

PS：SNORD78、SNORD51 and U6 primers were purchased from RIBOBIO.

**Supplementary table 4. Primers used for 3’RACE and in vitro transcription**

| **Application** | **Name** | **Sequence (5’->3’)** |
| --- | --- | --- |
| 3’-Full RACE Core Set with PrimeScript™ RTase | Outer Primer | TACCGTCGTTCCACTAGTGATTT |
|  | Inner Primer | CGCGGATCCTCCACTAGTGATTTCACTATAGG |
|  | 3’RACE Adaptor | Sequence was produced by Takara。 |
|  | Gene Specific Primer1 | GCCCTGTATCTTGGCTACTT |
|  | Gene Specific Primer2 | CCTCCAGTTCAGGTTCTGTT |
|  | Gene Specific Primer3 | GTGCAGCATTGAAAGGCCTT |
| In Vitro Transcription | SNORD51 primers | F: TAATACGACTCACTATAGGGAGAGTTGCATGATGAATAAAATCAAAT |
|  |  | R: AATCAGAAAAAAGCAAATCCATCAC |

**Supplementary table 5.** **Antibodies used for western blotting, RIP, IF and ChIP**

| **Protein** | **Application** | **Antibody** | **Origin** | **Dilution** | **Observed**  **Molecular weight** |
| --- | --- | --- | --- | --- | --- |
| KHDRBS1 | IP, WB, IF | 10222-1-AP, Proteintech | Rabbit | WB: 1:1000, IF: 1:50, IP: 2.0μg for IP | 68kDa |
| WDR33 | IP, IF | 22614-1-AP, Proteintech | Rabbit | IP: 2μg for IP, IF: 1:50 | 170kDa |
| ZBED6 | WB, IP | YT6573, Immunoway | Rabbit | IP: 2μg for IP,  WB: 1:1000 | 115kDa |
| β-actin | WB | 66009-1-Ig, Proteintech | Mouse | WB: 1:10000 | 42kDa |
| IgG | IP |  |  |  |  |
| G6PD | WB | 25413-1-AP, Proteintech | Rabbit | WB: 1:4000 | 59kDa |
| PGD | WB | 14717-1-AP, Proteintech | Rabbit | WB: 1:4000 | 53kDa |
| Goat anti-mouse IgG (H+L), HRP conjugate | WB | SA00001-1, Proteintech | Goat | WB: 1:10000 | None |
| Goat anti-rabbit IgG (H+L), HRP conjugate | WB | SA00001-2, Proteintech | Goat | WB: 1:10000 | None |
| Alexa-Fluor-488-labeled Goat anti-Rabbit IgG (H+L) | IF | A0423, [Beyotime](https://www.beyotime.com/index.htm) | Goat | IF: 1: 200 | None |

| **Gene** | **Target Sequence (5’->3’)** |
| --- | --- |
| sh-KHDRBS1#1 | GCTGCTGACGGCAGAAATTGA |
| sh-KHDRBS1#2 | GGACCACAAGGGAATACAATC |
| sh-KHDRBS1#3 | GGTGCAAAGATCTCTGTATTG |
| sh-KHDRBS1#4 | GGATCTGCATGTCTTCATTGA |
| sh-ZBED6#1 | GCAAGGCATTCACCTCATTGG |
| sh-ZBED6#2 | GCTTTAATTCCTGGAACTAGA |
| sh-ZBED6#3 | GGACCTTAGTGACTCTGATTC |
| sh-ZBED6#4 | GCTAAAGACTGTTTGATAACC |
| sh-WDR33#1 | GCAAGCAATGCAACAGCTTAC |
| sh-WDR33#2 | GCACATAAGGAGGCGATTAGA |
| sh-WDR33#3 | GCATGGTGCTGATGTGAAATG |
| sh-WDR33#4 | GCCAATCAAGTTCTGGGATCC |

**Supplementary table 6. shRNA used for transfection**

**Supplementary table 7. Primers used for ChIP experiments**

| **Gene** | **Binding or Control sites** | **Sequence (5’->3’)** | **Product Size(bp)** | **Annealing temperature (**°C) |
| --- | --- | --- | --- | --- |
| G6PD | PCR1 | F: CGATAGAGCGCAGTCAAGTTTG | 243 | 58.5 |
|  |  | R: CCGAAACTTCCCCCGGAC |  |  |
|  | Control | F: CGACAGAGTGAGACTGGGTC | 105 | 59.7 |
|  |  | R: TCTTGACCTTGTCATCCGCC |  |  |
| PGD | PCR1 | F: CCTAGATGACATTGCCCCGG | 132 | 59.5 |
|  |  | R: TCCTGCGTGAGTTGCTATGG |  |  |
|  | Control | F: GGTGGGTGGATCACTTGAGG | 168 | 58.9 |
|  |  | R: CCTCCCAGGTTCAAGAGATT |  |  |

**Supplementary table 8. Probes for EMSA**

| **Probe** | **Sequence (5’->3’)** |
| --- | --- |
| SNORD51 | GUUGCAUGAUGAAUAAAAUCAAAUCACCAU |
| 3’UTR of ZBED6 pre-mRNA | GUUUUUCUGUUUCCUUAAAAACCCUGAUGCUUAAU |
