# Supplementary material for: KHDRBS1 regulates the pentose phosphate pathway and malignancy of GBM through SNORD51-mediated polyadenylation of ZBED6 pre-mRNA
Source: Cell Death Dis. 2024 Nov 8;15(11):802. doi: 10.1038/s41419-024-07163-x (PMC11549417; doi:10.1038/s41419-024-07163-x)
Supplement: Supplementary file 2 — Supplemental methods [file 41419_2024_7163_MOESM2_ESM.docx]

**Supplementary Methods**

**Clinical Specimens**

Normal brain tissues (NBTs) of patients (n = 9) with traumatic brain trauma in neurosurgery at Shengjing Hospital affiliated to China Medical University were selected as negative control group, and tissues with postoperative pathological examination as glioma in patients with brain tumors were selected as experimental group. Glioma are classified according to the WHO classification into low-grade (WHO 1–2, n = 9) and high-grade glioma (WHO 3–4, n = 9). This study was confirmed by the Ethics Committee of Shengjing Hospital affiliated to China Medical University, and was approved by the patients and the families with informed consent obtained. Glioma inclusion criteria: (1) age superior to 18 years; (2) first onset, imaging (CT, MRI) identified intracranial mass lesions, hospitalization for surgical treatment and postoperative pathology diagnosis; (3) without radiotherapy, chemotherapy, and other treatments before surgery. Glioma exclusion criteria: (1) combined with hematologic disorders; (2) combined with other malignant tumors; (3) combined with other organs abnormal function; (4) combined with immune system diseases or connective tissue lesions. Negative control group inclusion criteria: (1) brain trauma; (2) without related diseases such as intracranial tumors, cerebral hemorrhage, and cerebral infarction before injury. Negative control group exclusion criteria: (1) other serious underlying diseases such as: coronary heart disease, cirrhosis, renal insufficiency; (2) other tumors. All the tissue samples were immediately frozen in liquid nitrogen after surgical resection and stored in liquid nitrogen until use.

**Cell culture**

Human embryonic kidney 293T (HEK-293T) cells and human GBM cell lines (U251 and U373) were purchased from the Shanghai Institutes for Biological Sciences Cell Resource Center and cultured in Dulbecco’s modified Eagle’s medium (Hyclone, UT, USA) supplemented with 10% fetal bovine serum (TBD, Tianjin, China). Normal human astrocytes were purchased from Shanghai Zeye Biotechnology and cultured in Roswell Park Memorial Institute-1640 medium (Hyclone, UT, USA) supplemented with 10% fetal bovine serum (TBD, Tianjin, China) and 1% penicillin-streptomycin solution (Solarbio, Beijing, China). All cells were maintained in an incubator at 37°C and 5% CO_2_.

**RNA extraction and quantitative real-time PCR**

According to the manufacturer’s instructions, RNA from cells and tissues was extracted by using the Trizol reagent (Life Technologies Corporation, CA, USA). The concentration and quality of extracted RNA were measured by using the Nanodrop SpectroPhotometer (ND-100, Thermo Fisher Scientific, MA, USA). The One-Step SYBR PrimeScript RT-PCR kit (Takara, Kyoto, Japan), Bulge-Loop miRNA qRT-PCR Starter kit (RIBOBIO, Guangzhou, China) and LightCycler 96 instrument (Roche, Basel, Switzerland) were used to detect the RNA relative expression of KHDRBS1, ZBED6 and SNORD51. The primers were provided in Table S3. β-actin and U6 were used as internal references. The relative quantification of above was calculated to the 2^-ΔΔCt^ value.

**Western blot**

The collected cells were lysed on ice with RIPA buffer (Beyotime, Jiangsu, China) supplemented with PMSF (Beyotime, Jiangsu, China) for 50 min, and then centrifuged at 17,000×g for 45min at 4°C. The supernatant was extracted and the concentration and quality of proteins were detected by BCA kit (Takara, Kyoto, Japan). After SDS-PAGE electrophoresis, proteins were transferred onto PVDF membranes. 5% BSA was used to blocked the PVDF membranes at room temperature for 2h. The PVDF membranes was incubated by corresponding primary antibodies at 4°C for 12h. After three rinses with TBST, the secondary antibodies coupled with horseradish peroxidase were incubated for 2h at room temperature. And then they were illuminated with BeyoECL Star using Tanon-5200 according to instructions. The primary antibodies were shown in Table S5.

**FISH**

According to the manufacturer’s instructions, the SNORD51 probe (Cy3-labeled, GenePharma, Suzhu, China) and the RNA FISH kit (SA-Biotin System, GenePharma, Suzhou, China) were used to detect the distribution of SNORD51 in U251 and U373 cells. The SNORD51 probe was shown in Table S3.

**Cell transfection**

The vectors with the full-length sequence of SNORD51 and ZBED6 (SNORD51(+) and ZBED6(+)), as well as their respective empty vectors (SNORD51(+)NC and ZBED6(+)NC), were constructed by GenePharma (Suzhou, China). The short hairpin RNAs against KHDRBS1 and ZBED6 (KHDRBS1(-) and ZBED6(-)), as well as their corresponding nonspecific vectors (KHDRBS1(-)NC and ZBED6(-)NC) were constructed GenePharma (Suzhou, China). The antisense oligonucleotide targeting SNORD51 was purchased from RIOBIO (Guangzhou, China). The above vectors and antisense oligonucleotide were transfected into U251 and U373 cells using Lipofectamine 3000 (Invitrogen, CA, USA). According to the manufacturer’s instructions, the stably transfected cell lines were maintained by G418 and puromycin (Solarbio, Beijing, China). Resistant cell clones were established around approximately 4 weeks. For details, see Table S3, 6 and Supplementary Fig. 9.

**Immunofluorescence**

Cells were cultured onto confocal dishes (Biosharp, Beijing, China), fixed by 4% paraformaldehyde (Biosharp, Beijing, China) for 15min at room temperature and then washed 3 times with PBS. Next, 0.2% Triton X-100 (Solarbio, Beijing, China) was added for 10min at room temperature, then blocked with 5% BSA (Solarbio, Beijing, China). The cells were incubated with specific primary antibodies at 4°C overnight. The next day, the cells were washed 3 times with PBST and then incubated with the Alexa-Fluor-488-labeled Goat anti-Rabbit IgG(H+L) (Beyotime, Jiangsu, China) at room temperature for 2h. And after three washes with PBS, the cells were stained with DAPI (Solarbio, Beijing, China) for 5 min at room temperature under photophobic condition. The cells were observed under a laser scanning confocal microscope (LSCM). The antibodies used were provided in Table S5.

**Cell proliferation assay**

1×10^4^ cells seeded in 96-well (Corning, NJ, USA) were cultured overnight at 37°C with 5% CO_2_. 10 μl of CCK-8 reagent (DOJINDO, Shanghai, China) was added to each well and then cells were cultured for 2h the next day. Absorbance was measured at a wavelength of 450nm using SpectraMax M5 microplate reader (Molecular Devices, USA).

**Cell migration assay**

Cell migration assays was performed using the HoloMonitor M4 culture system (Phase Holographic Imaging PHI AB, SE) according to the manufacturer’s protocols. The cells of each group were inoculated into a six-well plate at a concentration of 2×10^4^ cells/ml. After the cells were attached to the petri dish, they were placed on the HoloMonitor M4 culture system and set for imaging for 8h at 1h intervals. For each experimental group, we show the last image frame and the cell movements. At the start of the analysis 5 visually identifiable cells in each experimental set were selected for tracking. Their movements are displayed in spatial X-Y plots.

**Cell invasion assay**

The cells were diluted with serum-free medium and added to the upper chambers of transwell chambers (Corning, NY, USA), and the chambers were placed in 24-well plates (Corning, NY, USA) after each well adding 500 μl medium supplemented with serum. The cells were maintained in an incubator at 37°C and 5% CO_2_ for 48h. Then the cells were fixed on the lower layer of transwell chambers by 4% paraformaldehyde (Biosharp, Beijing, China) for 15min at room temperature, then the cells were washed with PBS and stained with Giemsa stain solution (Leagene Biotechnology, Beijing, China). The cells were observed under the microscope and counted by Image J software.

**RNA stability measurement**

RNA synthesis in the cells was inhibited by adding actinomycin D (APE×BIO Technology, TX, USA) into the medium and total RNA was extracted from the cells by Trizol reagent (Life Technologies Corporation, CA, USA) at different time points. The RNAs of corresponding genes were analyzed by qRT-PCR. Compared with zero time, the half-life of RNA was determined by its level decreasing to 50% at a certain time point.

**RIP Assay**

According to the manufacturer’s instructions, the interaction of KHDRBS1 with SNORD51 and WDR33 with SNORD51 was detected by the RNA-binding protein immunoprecipitation kit (BersinBio, Guangzhou, China). The KHDRBS1 and WDR33 antibodies (Proteintech, Chicago, IL, USA) were used for the RIP assay and IgG was used as a negative control. The cell lysate was incubated with compounds of RIP buffer, magnetic beads, and antibody. Then, the compound was incubated with proteinase K, and immunoprecipitated RNA was isolated. The RNA concentration was measured by Nanodrop Spectrophotometer (ND-100, Thermo, USA). Lastly, the purified RNA was analyzed by qRT-PCR to demonstrate the presence of the binding targets.

**RNA-Pull down assay**

According to the manufacturer’s instructions, the interaction of KHDRBS1 with SNORD51 and SNORD51 with WDR33 was detected by RNA-Pull down kit (BersinBio, Guangzhou, China). In brief, biotin-labeled SNORD51 was synthesized, and incubated with the cell lysates for 4h. Then, the protein combined with biotin-labeled SNORD51 was pull down with sreptavidin magnetic beads after incubation overnight. The bead-RNA-protein mixture was collected by low-speed centrifuge, and eluted through Handee spin columns. Wash the bead-RNA-protein mixture with the boiled SDS buffer. The retrieved proteins were detected by western blot with **β**-actin as the control.

**Rapid amplification of cDNA end and Sanger sequencing**

In this study, the 3’-Full RACE Core Set with PrimeScript^TM^ Tase (Takara, Kyoto, Japan) was used to detect the 3’UTR of ZBED6, and specific primers were designed to amplify the 3’UTR of ZBED6. The final amplified product was purified by using the DNA gel purification kit (Accurate Biology, Hunan, China) followed by Sanger sequencing. Details were shown in Supplementary figures and tables.

**Chromatin immunoprecipitation assay**

The Chromatin immunoprecipitation (ChIP) assay was performed on U251 and U373 cells using Simple ChIP Enzymatic Chromatin IP Kit (Cell signaling Technology, Danvers, Massachusetts, USA) according to the manufacturer’s protocol. GBM cells were cross-linked with formaldehyde for 10min and terminated with glycine. Cells were harvested in lysis buffer and micrococcal nuclease was used to digest the chromatin. 2% lysates were used as an input reference control and stored at -20℃, and other lysates were incubated with anti-ZBED6 antibody or normal IgG antibody with rotation. DNA crosslinks were reversed by NaCl and proteinase K and the ChIP DNA was finally purified. Details were shown in Supplementary figures and tables.

**Luciferase reporter assay**

The assays were performed 48h after transfection the indicated constructs into 2.4×10^4^ 293T cells per well seeded into 96-well plates. The cells were analyzed by the luciferase assays using the dual-luciferase reporter assays system. The relative luciferase activity was expressed as the ratio of firefly luciferase activity to renilla luciferase activity.

**Tumor xenograft in nude mice**

For in vivo study, Four-week-old athymic nude mice (BALB/c) were purchased from the Beijing HFK Bioscience (Beijing, China). All the animal experiments were performed following the Animal Welfare Act and approved by the Ethics Committee of China Medical University. The nude mice were divided into five groups: Control group, KHDRBS1(-) group, SNORD51(-) group, ZBED6(+) group and KHDRBS1(-) + SNORD51(-) + ZBED6(+) group. For subcutaneous implantation, the stable transfected and expression cells were selected, and 3×10^5^ cells were injected subcutaneously under right axilla area. The volumes of tumor were measured every 5 days according to the formula: mm^3^ = length × width^2^ / 2. At 45 days after subcutaneously injection, the mice were sacrificed and the tumors were separated. As for survival study, 3×10^5^ cells were injected into the right striatum. The number of survival mice was recorded every day and survival analysis was performed according to Kaplan-Meier survival curve.
