# Supplementary material for: KHDRBS1 regulates the pentose phosphate pathway and malignancy of GBM through SNORD51-mediated polyadenylation of ZBED6 pre-mRNA
Source: Cell Death Dis. 2024 Nov 8;15(11):802. doi: 10.1038/s41419-024-07163-x (PMC11549417; doi:10.1038/s41419-024-07163-x)
Supplement: Supplementary file 3 — Original Data File [file 41419_2024_7163_MOESM3_ESM.docx]

Fig. 1A

KHDRBS1 β-actin







Fig. 1C

KHDRBS1 β-actin


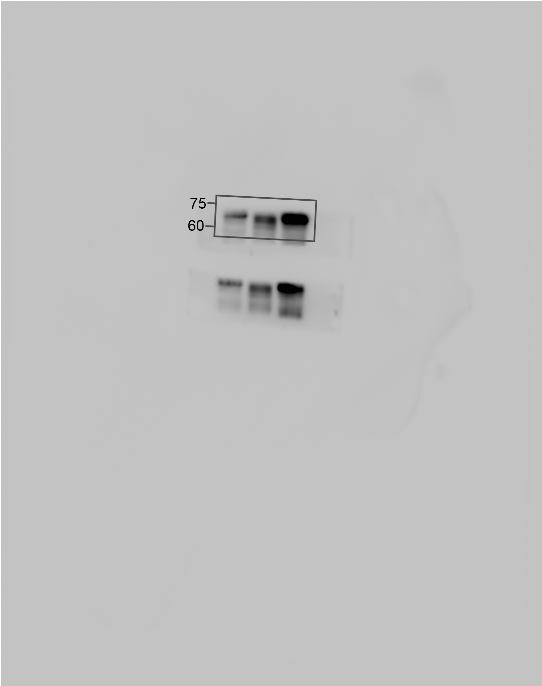

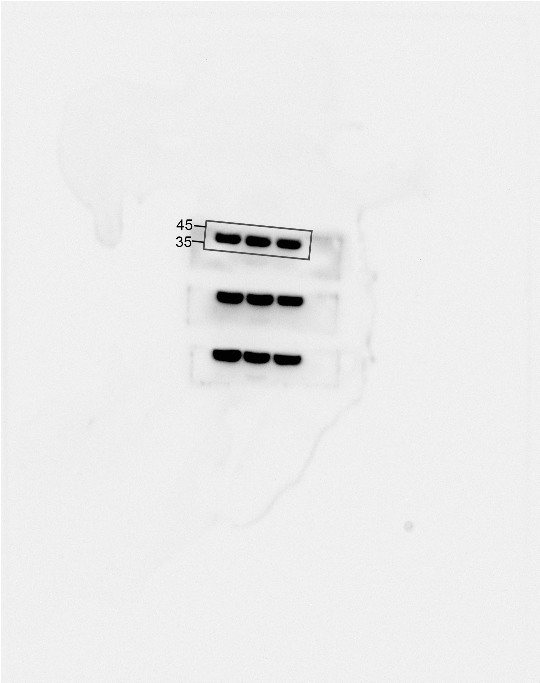


Fig. 1G

G6PD (U251) β-actin (U251)







G6PD (U373) β-actin (U373)







Fig. 2G

G6PD (U251) β-actin (U251)







G6PD (U373) β-actin (U373)







Supplementary fig. 3A

G6PD (U251) β-actin (U251)







G6PD (U373) β-actin (U373)







Fig. 3C

KHDRBS1 (RNA Pull-down) β-actin (RNA Pull-down)







Fig. 5C

ZBED6 β-actin







Fig. 5D

ZBED6 β-actin







Fig. 5G

G6PD (U251) β-actin (U251)







G6PD (U251) β-actin (U373)







Fig. 6C

WDR33 (RNA Pull-down) β-actin (RNA Pull-down)







Fig. 6G

WDR33 (Immunodepletion)





Fig. 6H

ZBED6 labeled probe (EMSA)


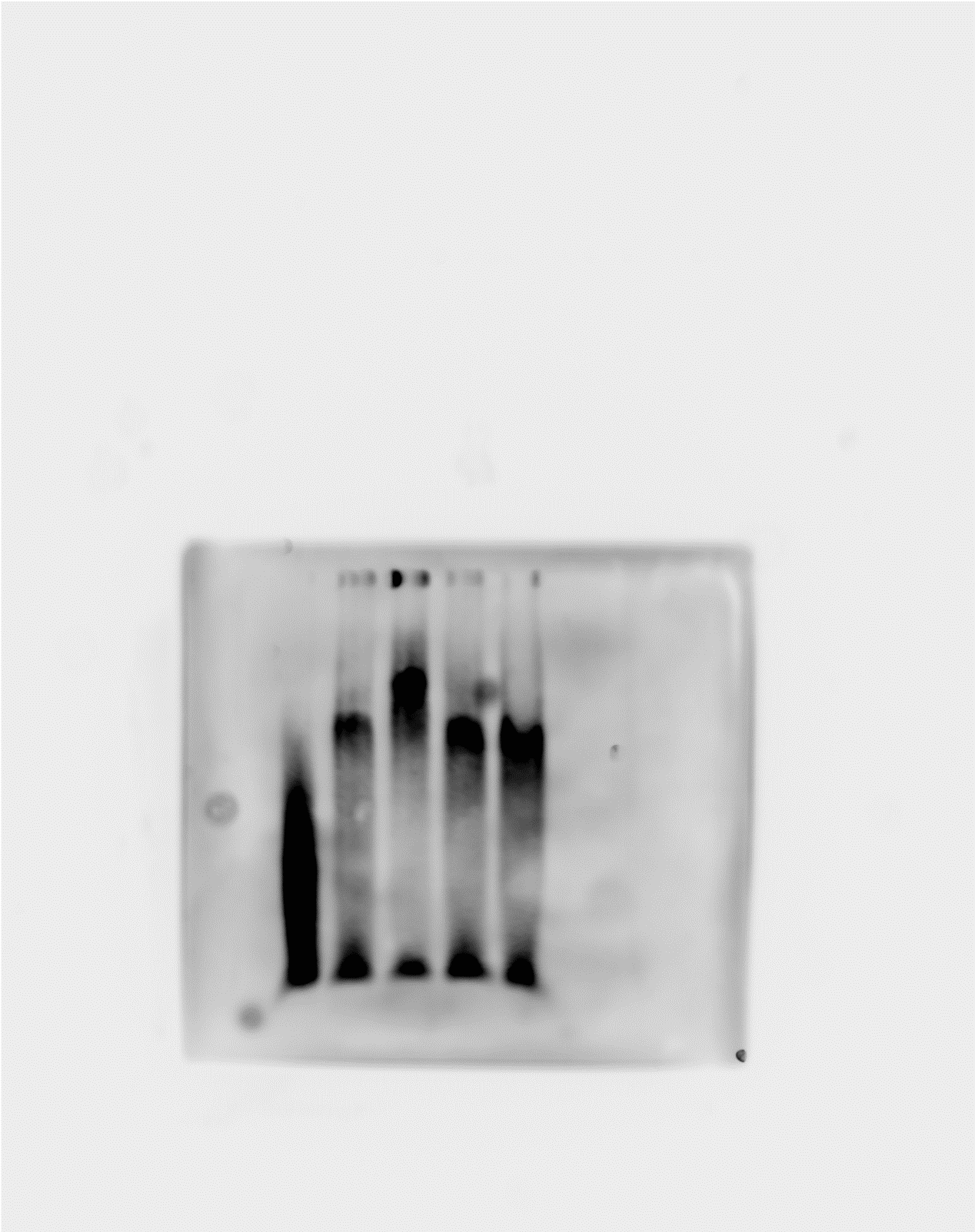


Fig. 7C

G6PD (U251) β-actin (U251)







G6PD (U373) β-actin (U373)







Supplementary fig. 8B

KHDRBS1 (U251) β-actin (U373)


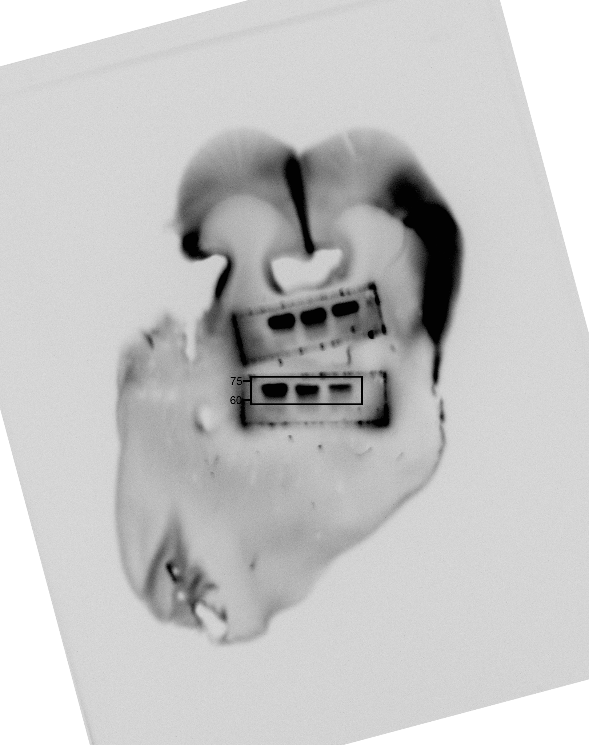

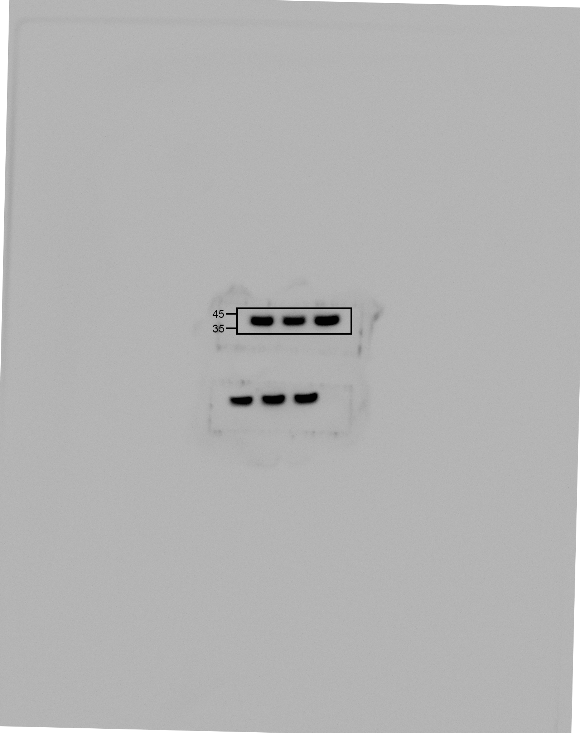


KHDRBS1 (U373) β-actin (U373)


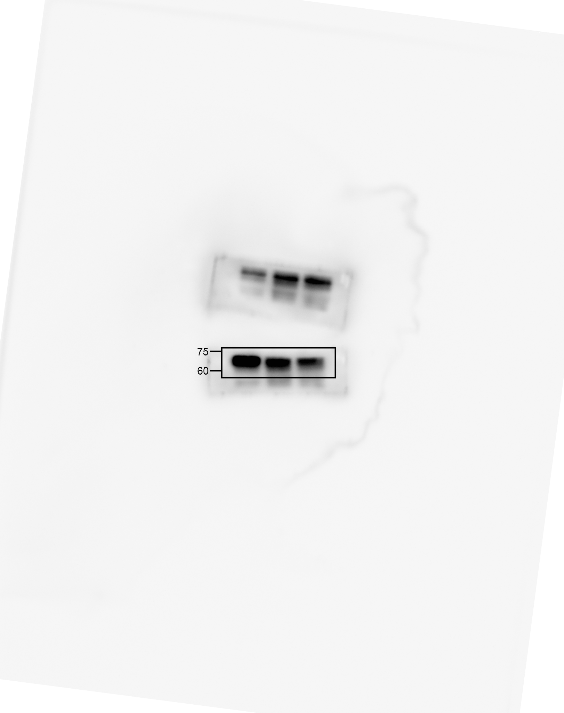

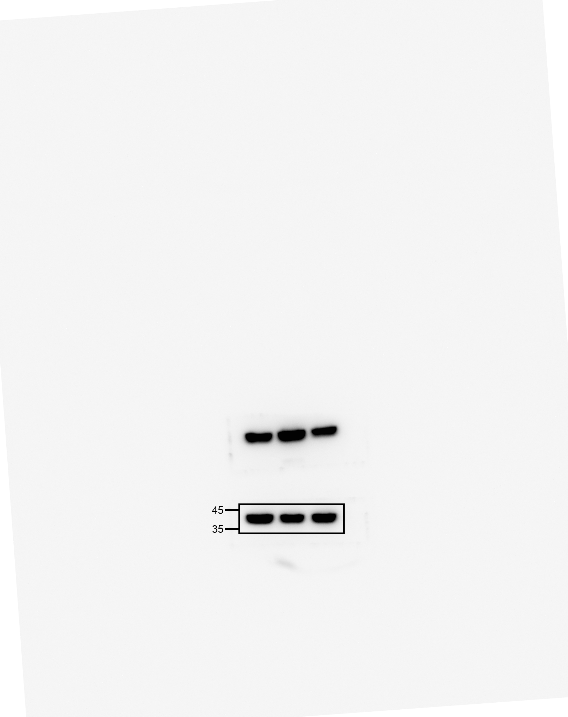


Supplementary fig. 8D

WDR33 (U251) β-actin (U251)


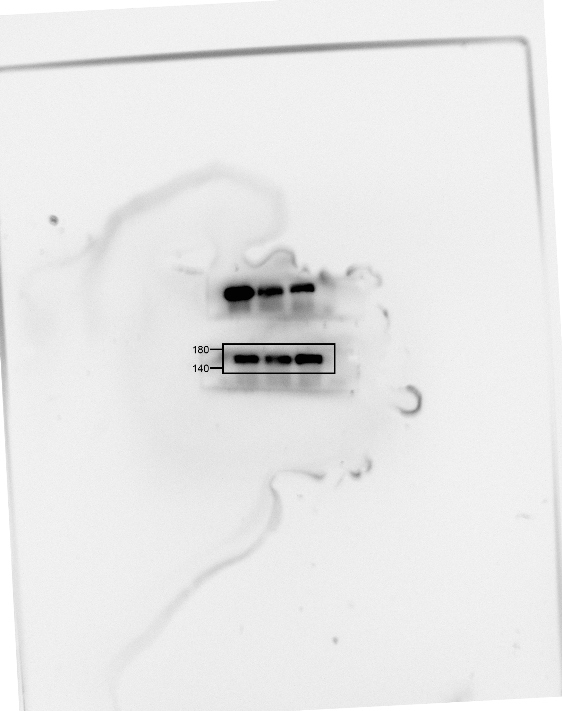

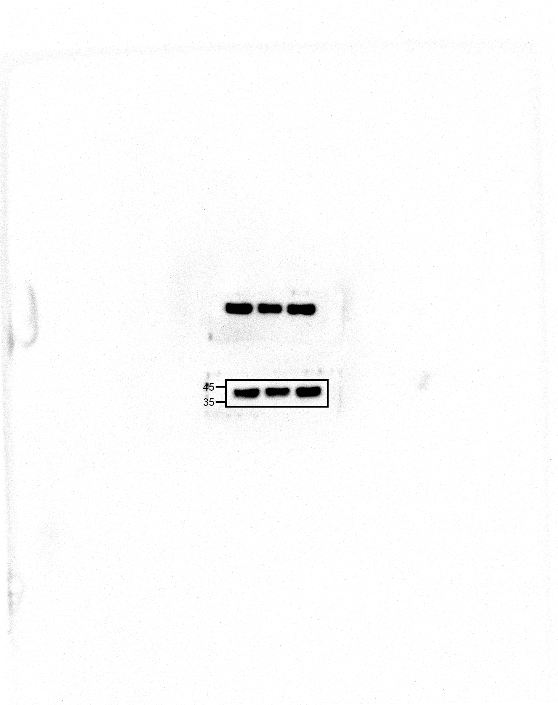


WDR33 (U373) β-actin (U373)


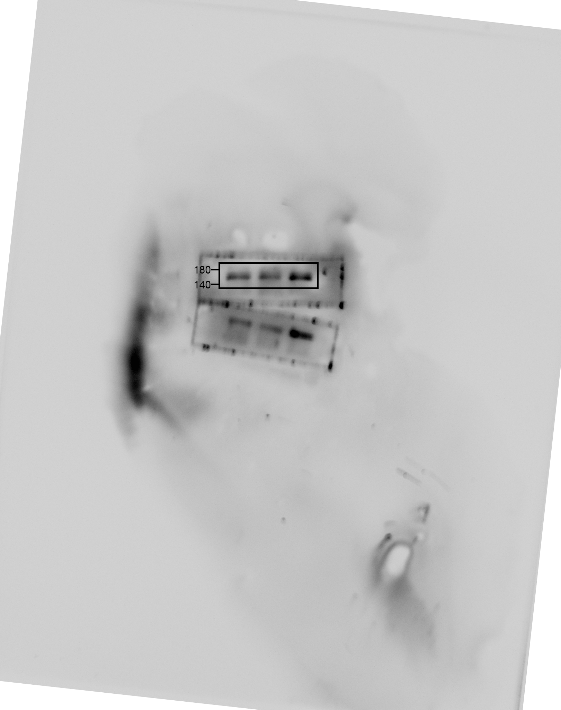

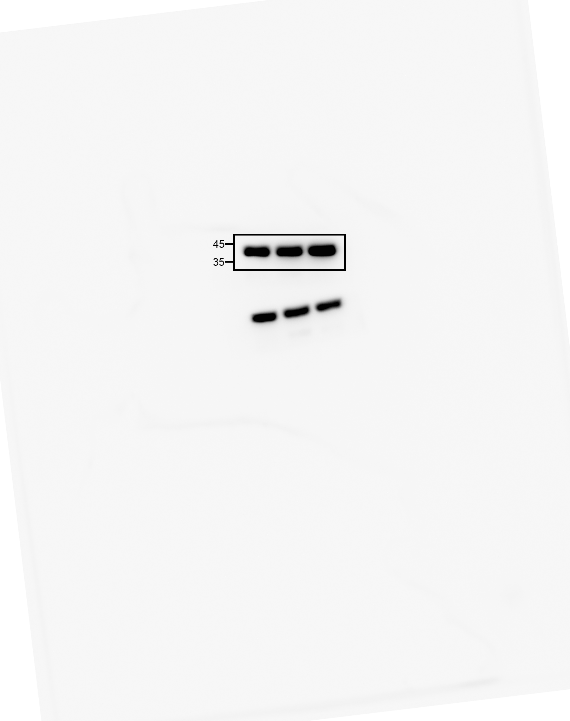


Supplementary fig. 8F

ZBED6 (U251) β-actin (U251)




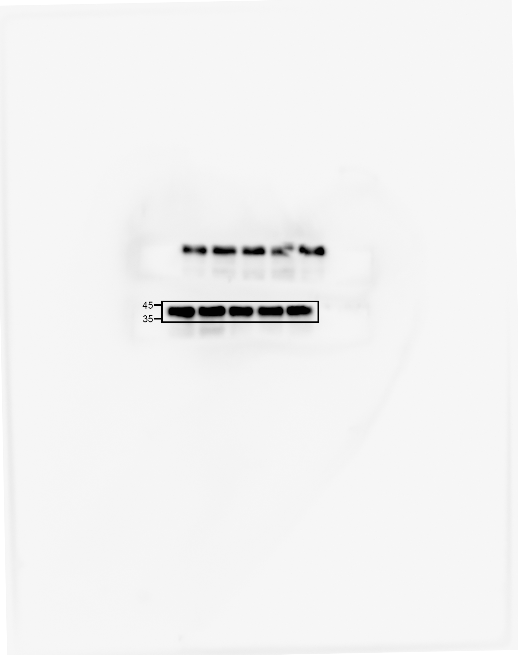


ZBED6 (U373) β-actin (U373)




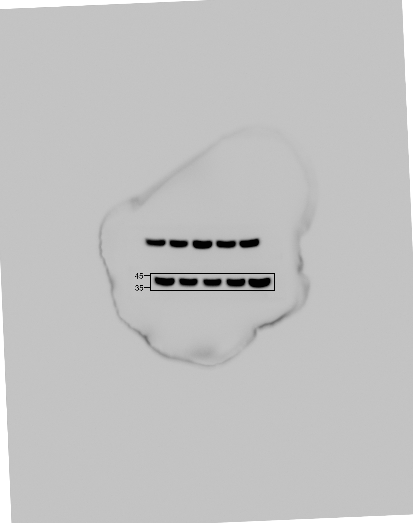


Supplementary fig. 9C

6PGD (U251) β-actin (U251)







6PGD (U373) β-actin (U373)


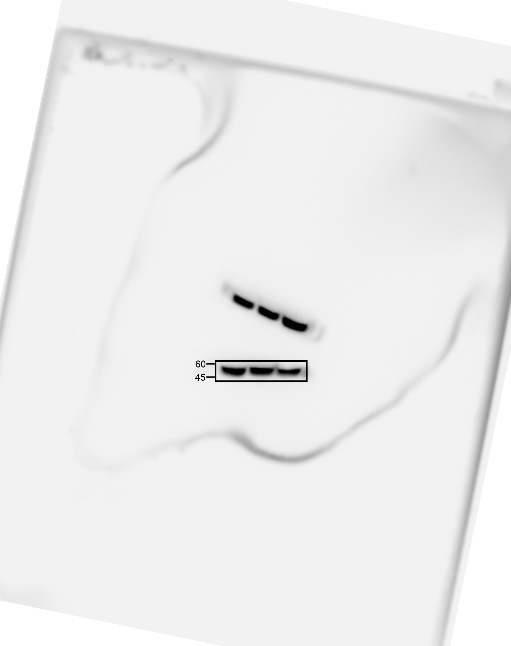




Supplementary fig. 9D

6PGD (U251) β-actin (U251)







6PGD (U373) β-actin (U373)







Supplementary fig. 10A

6PGD (U251) β-actin (U251)







6PGD (U373) β-actin (U373)







Supplementary fig. 10B

6PGD (U251) β-actin (U251)







6PGD (U373) β-actin (U373)







Supplementary fig. 10C

6PGD (U251) β-actin (U251)







6PGD (U373) β-actin (U373)
